# Generalised approach to modelling a three-tiered microbial food-web

**DOI:** 10.1016/j.mbs.2017.07.005

**Published:** 2017-09

**Authors:** T. Sari, M.J. Wade

**Affiliations:** a Irstea, UMR Itap, Montpellier, France & Université de Haute Alsace, Laboratoire de Mathématiques, Mulhouse, France; b School of Civil Engineering and Geosciences, Newcastle University, Newcastle-upon-Tyne NE1 7RU, United Kingdom

**Keywords:** Mathematical modelling, Dynamical systems, Stability theory, Microbial ecology, Anaerobic digestion

## Abstract

The complexity of the anaerobic digestion process has motivated the development of complex models, such as the widely used Anaerobic Digestion Model No. 1. However, this complexity makes it intractable to identify the stability profile coupled to the asymptotic behaviour of existing steady-states as a function of conventional chemostat operating parameters (substrate inflow concentration and dilution rate). In a previous study this model was simplified and reduced to its very backbone to describe a three-tiered chlorophenol mineralising food-web, with its stability analysed numerically using consensus values for the various biological parameters of the Monod growth functions. Steady-states where all organisms exist were always stable and non-oscillatory. Here we investigate a generalised form of this three-tiered food-web, whose kinetics do not rely on the specific kinetics of Monod form. The results are valid for a large class of growth kinetics as long as they keep the signs of their derivatives. We examine the existence and stability of the identified steady-states and find that, without a maintenance term, the stability of the system may be characterised analytically. These findings permit a better understanding of the operating region of the bifurcation diagram where all organisms exist, and its dependence on the biological parameters of the model. For the previously studied Monod kinetics, we identify four interesting cases that show this dependence of the operating diagram with respect to the biological parameters. When maintenance is included, it is necessary to perform numerical analysis. In both cases we verify the discovery of two important phenomena; i) the washout steady-state is always stable, and ii) a switch in dominance between two organisms competing for hydrogen results in the system becoming unstable and a loss in viability. We show that our approach results in the discovery of an unstable operating region in its positive steady-state, where all three organisms exist, a fact that has not been reported in a previous numerical study. This type of analysis can be used to determine critical behaviour in microbial communities in response to changing operating conditions.

## Highlights

• A three species microbial food-web is analysed to determine the existence and stability of steady-states.
• A generalised form of the model reduces the reliance on numerical assumptions.
• The analysis reveals the existence of unstable operating regions previously unobserved.
• The methodology provides opportunities for microbiologists to test the effect of species characteristics on the properties of the food-web.

## 1 Introduction

The mathematical modelling of engineered biological systems has entered a new era in recent years with the expansion and standardisation of existing models aimed at collating disparate components of these processes and provide scientists, engineers and practitioners with the tools to better predict, control and optimise them [23]. In engineered biological systems, mechanistic modelling reached consensus with the development of the Activated Sludge Models [9], [10] for wastewater treatment processes, followed by the Anaerobic Digestion Model No. 1 (ADM1) [12] a few years later. The development of ADM1 was enabled largely due to the possibilities for better identification and characterisation of functional microbial groups responsible for the chemical transformations within anaerobic digesters. It describes a set of stoichiometric and kinetic functions representing the standard anaerobic processes, remaining the scientific benchmark to the present day. However, there has been a growing awareness that the model should take advantage of improved empirical understanding and extension of biochemical processes included in its structure, to acquire a better trade-off between model realism and complexity [13].

The full ADM1 model is highly parameterised with a large number of physical, chemical and biological processes described by numerous state variables and algebraic expressions. Whilst suitable for dynamic simulation, more rigorous mathematical analysis of the model is difficult. To the authors knowledge, only numerical investigations are available [3]. Due to the analytical intractability of the full ADM1, work has been made towards the construction of simpler models that preserve biological meaning whilst reducing the computational effort required to find mathematical solutions of the model equations [7], [8].

The most common models used to describe microbial systems are two-tiered models, which take the form of a cascade of two biological reactions where one substrate is consumed by one microorganism to produce a product that serves as the main limiting substrate for a second microorganism. When the second organism has no feedback on the first organism, the system is known as commensalistic [16], [20]. The system has a cascade structure and the number of steady-states and their (mathematical) stability as a function of model inputs and parameters may be investigated [1], [2], [19]. When the growth of the first organism is affected by the substrate produced by the second organism the system is known as syntrophic. For instance, if the first organism is inhibited by high concentrations of the product, the extent to which the first substrate is degraded by the first organism depends on the efficiency of the removal of the product by the second organism. The mathematical analysis of such a model is more delicate than for commensalistic models, (see for instance early work by [4], [14], [15], [27] and the more recent papers [5], [11], [17], [18], [22]). An important and interesting extension should be mentioned here: [25], [26] analysed an 8-dimensional mathematical model, which includes syntrophy and inhibition, both mechanisms considered by [2] and [6].

As an example of this for anaerobic digestion, a previous study investigated the effect of maintenance on the stability of a two-tiered ‘food-chain’ comprising two species and two substrates [28]. Maintenance is defined as the energy consumed by an organism that is used for all biological processes other than growth. In [28] and here, it is analogous to a first-order decay rate constant, or biomass death term. Although the authors were not able to determine the general conditions under which this four dimensional syntrophic consortium was stable, further work has shown that a model with generality can be used to answer the question posed, determining that the two-tiered food-chain is always stable when maintenance is included [18].

**Fig. 1:**
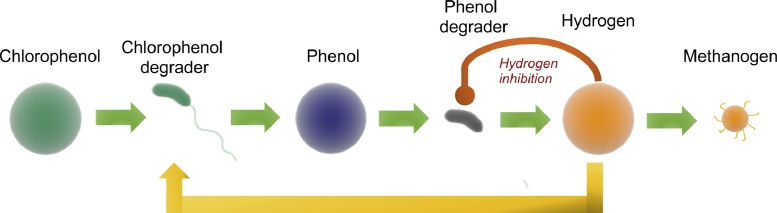
Schematic of the three-tier chlorophenol mineralising food-web indicating the flow and conversion of chemical substrates and products in the system.

More recently, the model described by [28] was extended by the addition of a third organism and substrate to create a three-tiered ‘food-web’ [24], as shown in Fig. 1. In this paper, the existence and stability of the steady-states were determined only numerically. Although the results were important in revealing emergent properties of this extended model, the motivation of this work is to give an analytical study of the model. Moreover, our analysis does not require that growth functions are of the specific form considered and are valid for a large class of growth functions. This is critical as it provides the means by which microbiologists can theoretically test the influence of the growth characteristics of organisms on the properties of the system and the interactions between multiple species.

Here, we pursue a generalised description and analysis of the model given by [24]. Chlorophenols are chemicals of importance due to their impact on the environment and to public health, their recalcitrance in food-webs and resistance to aerobic biodegradation via the oxygenase enzyme [21]. Although we consider the monochlorophenol isomer here, extension to multiple isomeric chlorophenols would be straightforward. It is important to note that, although the particular biological transformation provided is for chlorophenol mineralisation, the structure and methods employed are much more general and apply to any theoretical ecological interactions that may be hypothesised or observed in a microbial community. We therefore stress that this work can provide a good approach for analytically investigating the behaviour of microbial food-webs where numerical parameters are difficult to obtain or uncertain. Ultimately, we demonstrate here the advantage of a generalised approach for mathematical analysis of microbial ecological interactions from both a theoretical perspective and its potential if providing knowledge in applied research where these communities and processes are studied empirically.

The paper is organised as follows. In Section 2, we present a description of the model to be investigated, and its reduction in Section 3. Model assumptions and notations are provided in Section 4. In Section 5 we demonstrate the existence of the three steady-states and define four interesting cases for specific parameter values that are investigated using the solutions, whilst also indicating the regions of existence of the steady-states for the operating parameter values. We present results on the behaviour of the system whilst varying two control parameters in Section 6. In Section 7 we perform local stability analysis of the steady-states without maintenance and, in Section 8, we undertake a comprehensive numerical stability analysis of the four cases for both the model with and without maintenance. We show that our approach leads to the discovery of five operating regions in which one leads to the possibility of instability of the positive steady-state, where all three organisms exist, a fact that has not been reported by [24]. Indeed, we suggest that a stable limit-cycle can occur at the boundary with this region. Finally, in Section 9, we make comment on the role of the kinetic parameters used in the four example cases, in maintaining stability, which points to the importance of the relative aptitude of the two hydrogen consumers in sustaining a viable chlorophenol mineralising community. In the Appendix we describe the numerical method used in Section 8, give the assumptions on the growth functions we used and the proofs of the results.

## 2 The model

The model developed in [24] has six components, three substrate (chlorophenol, phenol and hydrogen) and three biomass (chlorophenol and phenol degraders, and a hydrogenotrophic methanogen) variables. The substrate and biomass concentrations evolve according to the six-dimensional dynamical of ODEs:

(1) $$\begin{array}{cc} \frac{dX_{\text{ch}}}{dt} & =-DX_{\text{ch}}+Y_{\text{ch}}f_{0}\left(S_{\text{ch}},S_{H_{2}}\right)X_{\text{ch}}-k_{\text{dec},\text{ch}}X_{\text{ch}} \end{array}$$

(2) $$\begin{array}{cc} \frac{dX_{\text{ph}}}{dt} & =-DX_{\text{ph}}+Y_{\text{ph}}f_{1}\left(S_{\text{ph}},S_{H_{2}}\right)X_{\text{ph}}-k_{\text{dec},\text{ph}}X_{\text{ph}} \end{array}$$

(3) $$\begin{array}{cc} \frac{dX_{H_{2}}}{dt} & =-DX_{H_{2}}+Y_{H_{2}}f_{2}\left(S_{H_{2}}\right)X_{H_{2}}-k_{\text{dec},H_{2}}X_{H_{2}} \end{array}$$

(4) $$\begin{array}{cc} \frac{dS_{\text{ch}}}{dt} & =D\left(S_{\text{ch},\text{in}}-S_{\text{ch}}\right)-f_{0}\left(S_{\text{ch}},S_{H_{2}}\right)X_{\text{ch}} \end{array}$$

(5) $$\begin{array}{ccc} \frac{dS_{\text{ph}}}{dt} & = & D\left(S_{\text{ph},\text{in}}-S_{\text{ph}}\right)+\frac{224}{208}\left(1-Y_{\text{ch}}\right)f_{0}\left(S_{\text{ch}},S_{H_{2}}\right)X_{\text{ch}}\\ & & -f_{1}\left(S_{\text{ph}},S_{H_{2}}\right)X_{\text{ph}} \end{array}$$

(6) $$\begin{array}{ccc} \frac{dS_{H_{2}}}{dt} & = & D\left(S_{H_{2},\text{in}}-S_{H_{2}}\right)+\frac{32}{224}\left(1-Y_{\text{ph}}\right)f_{1}\left(S_{\text{ph}},S_{H_{2}}\right)X_{\text{ph}}\\ & & -\frac{16}{208}f_{0}\left(S_{\text{ch}},S_{H_{2}}\right)X_{\text{ch}}-f_{2}\left(S_{H_{2}}\right)X_{H_{2}} \end{array}$$

where *S*_ch_ and *X*_ch_ are the chlorophenol substrate and degrader concentrations, *S*_ph_ and *X*_ph_ for phenol and $S_{H_{2}}$ and $X_{H_{2}}$ for hydrogen; *Y*_ch_, *Y*_ph_ and *Y*_H2_ are the yield coefficients, $224/208\left(1-Y_{\text{ch}}\right)$ represents the part of chlorophenol degraded to phenol, and $32/224\left(1-Y_{\text{ph}}\right)$ represents the part of phenol that is transformed to hydrogen. Growth functions take Monod form with hydrogen inhibition acting on the phenol degrader and represented in *f*_1_ (see (Eq. 7)) as a product inhibition term. In [24], this model is given in dimensionless form that significantly reduces the number of parameters describing the dynamics. In the analysis of the generalised model (Sections 5 and 7), we do not assume that the growth functions *f*_0_, *f*_1_ and *f*_2_ have the specific analytical expression shown. We will only assume that the growth functions satisfy properties that are listed in Section 4. Therefore, we cannot benefit from the dimensionless rescaling used by [24], because it uses some kinetics parameters of the specific growth functions (Eq. 7), while we work with general *unspecified* growth functions.

In Section 3 we consider another rescaling that does not use the kinetics parameters. For the numerical analyses given in Section 8, we return to an assumed set of Monod growth functions given by (Eq. 7), in order to directly compare with the results found by [24].

(7) $$\begin{array}{ccc} f_{0}\left(S_{\text{ch}},S_{H_{2}}\right) & = & \frac{k_{m,\text{ch}}S_{\text{ch}}}{K_{S,ch}+S_{\text{ch}}}\frac{S_{H_{2}}}{K_{S,H_{2},c}+S_{H_{2}}}\\ f_{1}\left(S_{\text{ph}},S_{H_{2}}\right) & = & \frac{k_{m,\text{ph}}S_{\text{ph}}}{K_{S,\text{ph}}+S_{\text{ph}}}\frac{1}{1+\frac{S_{H_{2}}}{K_{i,H_{2}}}}\\ f_{2}\left(S_{H_{2}}\right) & = & \frac{k_{m,H_{2}}S_{H_{2}}}{K_{S,H_{2}}+S_{H_{2}}} \end{array}$$

Here, apart from the four operating (or control) parameters, which are the inflowing concentrations *S*_ch, in_, *S*_ph, in_, $S_{H_{2},\text{in}}$ and the dilution rate *D*, that can vary, all others have biological meaning and are fixed depending on the organisms and substrate considered. We use the following notations in ((1), (2), (3), (4), (5), (6)):

$$\begin{array}{cc} & X_{0}=X_{\text{ch}},\;X_{1}=X_{\text{ph}},\;X_{2}=X_{H_{2}}\\ & S_{0}=S_{\text{ch}},\;S_{1}=S_{\text{ph}},\;S_{2}=S_{H_{2}}\\ & S_{0}^{\text{in}}=S_{\text{ch},\text{in}},\;S_{1}^{\text{in}}=S_{\text{ph},\text{in}},\;S_{2}^{\text{in}}=S_{H_{2},\text{in}}\\ & Y_{0}=Y_{\text{ch}},\;Y_{1}=Y_{\text{ph}},\;Y_{2}=Y_{H_{2}}\\ & Y_{3}=\frac{224}{208}\left(1-Y_{\text{ch}}\right),\;Y_{4}=\frac{32}{224}\left(1-Y_{\text{ph}}\right),\;Y_{5}=\frac{16}{208}\\ & a_{0}=k_{\text{dec},\text{ch}},\;a_{1}=k_{\text{dec},\text{ph}},\;a_{2}=k_{\text{dec},H_{2}} \end{array}$$

With these notations ((1), (2), (3), (4), (5), (6)) can be written as follows:

(8) $$\begin{array}{cc} \frac{dX_{0}}{dt} & =-DX_{0}+Y_{0}f_{0}\left(S_{0},S_{2}\right)X_{0}-a_{0}X_{0} \end{array}$$

(9) $$\begin{array}{cc} \frac{dX_{1}}{dt} & =-DX_{1}+Y_{1}f_{1}\left(S_{1},S_{2}\right)X_{1}-a_{1}X_{1} \end{array}$$

(10) $$\begin{array}{cc} \frac{dX_{2}}{dt} & =-DX_{2}+Y_{2}f_{2}\left(S_{2}\right)X_{2}-a_{2}X_{2} \end{array}$$

(11) $$\begin{array}{cc} \frac{dS_{0}}{dt} & =D\left(S_{0}^{\text{in}}-S_{0}\right)-f_{0}\left(S_{0},S_{2}\right)X_{0} \end{array}$$

(12) $$\begin{array}{cc} \frac{dS_{1}}{dt} & =D\left(S_{1}^{\text{in}}-S_{1}\right)+Y_{3}f_{0}\left(S_{0},S_{2}\right)X_{0}-f_{1}\left(S_{1},S_{2}\right)X_{1} \end{array}$$

(13) $$\begin{array}{cc} \frac{dS_{2}}{dt} & =D\left(S_{2}^{\text{in}}-S_{2}\right)+Y_{4}f_{1}\left(S_{1},S_{2}\right)X_{1}-Y_{5}f_{0}\left(S_{0},S_{2}\right)X_{0}-f_{2}\left(S_{2}\right)X_{2} \end{array}$$

Furthermore, we restrict our analysis to the case where we only have one substrate added to the system, such that: $S_{0}^{in}>0,$$S_{1}^{in}=0,$ and $S_{2}^{in}=0$. As shown in [24], the general case with $S_{1}^{in}>0,$ and $S_{2}^{in}>0$ present many important and interesting behaviours and deserves future work.

## 3 Model reduction

To ease the mathematical analysis, we can rescale the system ((8), (9), (10), (11), (12), (13)) using the following change of variables adapted from [18]:

$$\begin{array}{cc} & x_{0}=\frac{Y_{3}Y_{4}}{Y_{0}}X_{0},\;x_{1}=\frac{Y_{4}}{Y_{1}}X_{1},\;x_{2}=\frac{1}{Y_{2}}X_{2}\\ & s_{0}=Y_{3}Y_{4}S_{0},\;s_{1}=Y_{4}S_{1},\;s_{2}=S_{2} \end{array}$$

We obtain the following system:

(14) $$\begin{array}{cc} \frac{dx_{0}}{dt} & =-Dx_{0}+\mu_{0}\left(s_{0},s_{2}\right)x_{0}-a_{0}x_{0} \end{array}$$

(15) $$\begin{array}{cc} \frac{dx_{1}}{dt} & =-Dx_{1}+\mu_{1}\left(s_{1},s_{2}\right)x_{1}-a_{1}x_{1} \end{array}$$

(16) $$\begin{array}{cc} \frac{dx_{2}}{dt} & =-Dx_{2}+\mu_{2}\left(s_{2}\right)x_{2}-a_{2}x_{2} \end{array}$$

(17) $$\begin{array}{cc} \frac{ds_{0}}{dt} & =D\left(s_{0}^{\text{in}}-s_{0}\right)-\mu_{0}\left(s_{0},s_{2}\right)x_{0} \end{array}$$

(18) $$\begin{array}{cc} \frac{ds_{1}}{dt} & =-Ds_{1}+\mu_{0}\left(s_{0},s_{2}\right)x_{0}-\mu_{1}\left(s_{1},s_{2}\right)x_{1} \end{array}$$

(19) $$\begin{array}{cc} \frac{ds_{2}}{dt} & =-Ds_{2}+\mu_{1}\left(s_{1},s_{2}\right)x_{1}-\omega \mu_{0}\left(s_{0},s_{2}\right)x_{0}-\mu_{2}\left(s_{2}\right)x_{2} \end{array}$$

where the inflowing concentration is:

(20) $$s_{0}^{\text{in}}=Y_{3}Y_{4}S_{0}^{\text{in}},$$

the growth functions are:

(21) $$\begin{array}{ccc} & & \mu_{0}\left(s_{0},s_{2}\right)=Y_{0}f_{0}\left(\frac{s_{0}}{Y_{3}Y_{4}},s_{2}\right)\\ & & \mu_{1}\left(s_{1},s_{2}\right)=Y_{1}f_{1}\left(\frac{s_{1}}{Y_{4}},s_{2}\right)\\ & & \mu_{2}\left(s_{2}\right)=Y_{2}f_{2}\left(s_{2}\right) \end{array}$$

and

(22) $$\omega =\frac{Y_{5}}{Y_{3}Y_{4}}=\frac{1}{2\left(1-Y_{0}\right)\left(1-Y_{1}\right)}$$

The benefit of our rescaling is that it permits to fix in ((14), (15), (16), (17), (18), (19)) all yield coefficients to one except that denoted by *ω* and defined by (Eq. 22), and to discuss the existence and stability with respect to this sole parameter.

Using (Eq. 21) and the growth functions (Eq. 7), we obtain the model ((14), (15), (16), (17), (18), (19)) with the following Monod-type growth functions:

(23) $$\begin{array}{ccc} & & \mu_{0}\left(s_{0},s_{2}\right)=\frac{m_{0}s_{0}}{K_{0}+s_{0}}\frac{s_{2}}{L_{0}+s_{2}}\\ & & \mu_{1}\left(s_{1},s_{2}\right)=\frac{m_{1}s_{1}}{K_{1}+s_{1}}\frac{1}{1+s_{2}/K_{i}}\\ & & \mu_{2}\left(s_{2}\right)=\frac{m_{2}s_{2}}{K_{2}+s_{2}} \end{array}$$

where:

(24) $$\begin{array}{cc} & m_{0}=Y_{0}k_{m,\text{ch}},\;K_{0}=Y_{3}Y_{4}K_{s,\text{ch}},\;L_{0}=K_{S,H_{2},c}\\ & m_{1}=Y_{1}k_{m,\text{ph}},\;K_{1}=Y_{4}K_{s,\text{ph}},\;K_{i}=K_{i,H_{2}}\\ & m_{2}=Y_{2}k_{m,H_{2}},\;K_{2}=K_{S,H_{2}} \end{array}$$

For the numerical simulations we will use the nominal values in Table 1 given in [24].

**Table 1:** Nominal parameter values. We use units expressed in Chemical Oxygen Demand (COD) as used by [12], [24].

Parameters	Nominal values	Units
*k*_*m*, ch_	29	kgCOD_S_/kgCOD_X_/d
*K*_*S*, ch_	0.053	kgCOD/m^3^
*Y*_ch_	0.019	kgCOD_X_/kgCOD_S_
*k*_*m*, ph_	26	kgCOD_S_/kgCOD_X_/d
*K*_*S*, ph_	0.302	kgCOD/m^3^
*Y*_ph_	0.04	kgCOD_X_/kgCOD_S_
$k_{m,H_{2}}$	35	kgCOD_S_/kgCOD_X_/d
$K_{S,H_{2}}$	2.5$\times 10^{-5}$	kgCOD/m^3^
$K_{S,H_{2},c}$	1.0$\times 10^{-6}$	kgCOD/m^3^
$Y_{H_{2}}$	0.06	kgCOD_X_/kgCOD_S_
*k*_dec, i_	0.02	$d^{-1}$
$K_{I,H_{2}}$	3.5$\times 10^{-6}$	kgCOD/m^3^

## 4 Assumptions on the model and notations

Our study does not require that growth functions are of Monod type (Eq. 23). Actually, the results are valid for a more general class of growth functions satisfying the following conditions, which concur with those given by (Eq. 23):

H1 For all *s*_0_ > 0 and *s*_2_ > 0 then $0<\mu_{0}\left(s_{0},s_{2}\right)<+\infty$ and $\mu_{0}\left(0,s_{2}\right)=0,$$\mu_{0}\left(s_{0},0\right)=0$.
H2 For all *s*_1_ > 0 and *s*_2_ ≥ 0 then $0<\mu_{1}\left(s_{1},s_{2}\right)<+\infty$ and $\mu_{1}\left(0,s_{2}\right)=0$.
H3 For all *s*_2_ > 0 then $0<\mu_{2}\left(s_{2}\right)<+\infty$ and $\mu_{2}\left(0\right)=0$.
H4 For all *s*_0_ > 0 and *s*_2_ > 0,
  $$\frac{\partial \mu_{0}}{\partial s_{0}}\left(s_{0},s_{2}\right)>0,\;\frac{\partial \mu_{0}}{\partial s_{2}}\left(s_{0},s_{2}\right)>0.$$
H5 For all *s*_1_ > 0 and *s*_2_ > 0,
  $$\frac{\partial \mu_{1}}{\partial s_{1}}\left(s_{1},s_{2}\right)>0,\;\frac{\partial \mu_{1}}{\partial s_{2}}\left(s_{1},s_{2}\right)<0.$$
H6 For all *s*_2_ > 0, $\frac{d\mu_{2}}{ds_{2}}\left(s_{2}\right)>0$.
H7 The function $s_{2}\mapsto \mu_{0}\left(+\infty ,s_{2}\right)$ is monotonically increasing and the function $s_{2}\mapsto \mu_{1}\left(+\infty ,s_{2}\right)$ is monotonically decreasing.

The proof of the following result is standard and hence omitted.

Proposition 1 *For non-negative initial conditions, all solutions of the system (Eqs.* 14*–*19*) are bounded and remain non-negative for all t* > 0*.*

In the following lemma we define the functions *M*_0_(*y, s*_2_), *M*_1_(*y, s*_2_) and *M*_2_(*y*), which are the inverse functions of the functions *s*_0_↦*μ*_0_(*s*_0_, *s*_2_), *s*_1_↦*μ*_1_(*s*_1_, *s*_2_) and *s*_2_↦*μ*_2_(*s*_2_), respectively.

Lemma 1 *Let s*_2_ ≥ 0 *be fixed. There exists a unique function*

$$y\in \left[0,\mu_{0}\left(+\infty ,s_{2}\right)\right)\mapsto M_{0}\left(y,s_{2}\right)\in \left[0,+\infty \right),$$

*such that for s*_0_ ≥ 0, *s*_2_ ≥ 0 *and*$y\in [0,\mu_{0}\left(+\infty ,s_{2}\right)),$ *we have:*

(25) $$s_{0}=M_{0}\left(y,s_{2}\right)⟺y=\mu_{0}\left(s_{0},s_{2}\right)$$

*Let s*_2_ ≥ 0 *be fixed. There exists a unique function*

$$y\in \left[0,\mu_{1}\left(+\infty ,s_{2}\right)\right)\mapsto M_{1}\left(y,s_{2}\right)\in \left[0,+\infty \right),$$

*such that for s*_1_ ≥ 0, *s*_2_ ≥ 0 *and*$y\in [0,\mu_{1}\left(+\infty ,s_{2}\right)),$ *we have:*

(26) $$s_{1}=M_{1}\left(y,s_{2}\right)⟺y=\mu_{1}\left(s_{1},s_{2}\right)$$

*There exists a unique function*

$$y\in \left[0,\mu_{2}\left(+\infty \right)\right)\mapsto M_{2}\left(y\right)\in \left[0,+\infty \right),$$

*such that, for s*_2_ ≥ 0 *and*$y\in [0,\mu_{2}\left(+\infty \right))$ *we have:*

(27) $$s_{2}=M_{2}\left(y\right)⟺y=\mu_{2}\left(s_{2}\right)$$

**Fig. 2:**
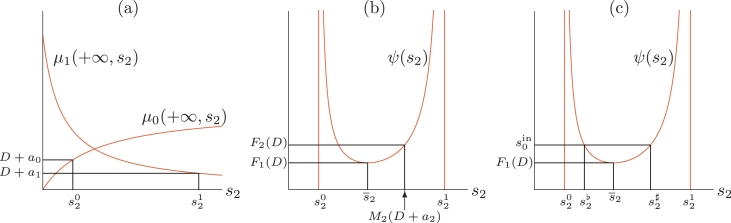
Graphical definitions. (a): $s_{2}^{0}$ and $s_{2}^{1}$. (b) : *ψ*(*s*_2_), ${\bar{s}}_{2},$*F*_1_(*D*) and *F*_2_(*D*). (c): $s_{2}^{♭}$ and $s_{2}^{♯}$.

Using the functions *M*_0_, *M*_1_ and *M*_2_ we define now the function *ψ*(*s*_2_, *D*), *F*_1_(*D*), *F*_2_(*D*) and *F*_3_(*D*). Let us observe first that by **H7**, for $D+a_{0}<\mu_{0}\left(+\infty ,+\infty \right)$ and $D+a_{1}<\mu_{1}\left(+\infty ,0\right)$ there exist unique values $s_{2}^{0}=s_{2}^{0}\left(D\right)$ and $s_{2}^{1}=s_{2}^{1}\left(D\right),$ see Fig. 2(a):

(28) $$\mu_{0}\left(+\infty ,s_{2}^{0}\right)=D+a_{0},\;\mu_{1}\left(+\infty ,s_{2}^{1}\right)=D+a_{1}$$

Assume that *ω* < 1. Let *D* be fixed such that $s_{2}^{0}\left(D\right)$ and $s_{2}^{1}\left(D\right)$ exist. We consider the function defined on $\left(s_{2}^{0}\left(D\right),s_{2}^{1}\left(D\right)\right)$ by:

(29) $$\begin{array}{ccc} & & s_{2}\in \left(s_{2}^{0}\left(D\right),s_{2}^{1}\left(D\right)\right)\mapsto \psi \left(s_{2},D\right)\\ & & \;=M_{0}\left(D+a_{0},s_{2}\right)+\frac{M_{1}\left(D+a_{1},s_{2}\right)+s_{2}}{1-\omega }, \end{array}$$

It should be noted that *ψ*(*s*_2_, *D*) > 0 for $s_{2}^{0}\left(D\right)<s_{2}<s_{2}^{1}\left(D\right)$. The function *ψ* together with the values $s_{2}^{0},$$s_{2}^{1}$ all depend on *D*. However, to avoid cumbersome notation we will use the more precise form *ψ*(*s*_2_, *D*), $s_{2}^{0}\left(D\right)$ and $s_{2}^{1}\left(D\right)$ only if necessary. From (Eq. 25), (Eq. 26) and (Eq. 28) we deduce that:

$$M_{0}\left(D+a_{0},s_{2}^{0}\right)=+\infty ,\;M_{1}\left(D+a_{1},s_{2}^{1}\right)=+\infty$$

Therefore, we have, see Fig. 2(b):

$$\lim_{s_{2}\to s_{2}^{0}}\psi \left(s_{2}\right)=\lim_{s_{2}\to s_{2}^{1}}\psi \left(s_{2}\right)=+\infty$$

Hence, the function *ψ*(*s*_2_), which is positive and tends to +∞ at the extremities of the interval $(s_{2}^{0},s_{2}^{1}),$ has a minimum value on this interval. We add the following assumption:

H8 The function *ψ* has a unique minimum ${\bar{s}}_{2}\left(D\right)$ on the interval $\left(s_{2}^{0}\left(D\right),s_{2}^{1}\left(D\right)\right)$ and $\frac{\partial \psi }{\partial s_{2}}\left(s_{2},D\right)$ is negative on $\left(s_{2}^{0}\left(D\right),{\bar{s}}_{2}\left(D\right)\right)$ and positive on $\left({\bar{s}}_{2}\left(D\right),s_{2}^{1}\left(D\right)\right),$ respectively.

**Fig. 3:**
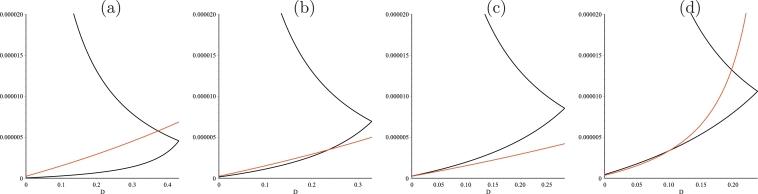
Graphs of $s_{2}^{0}\left(D\right)$ and $s_{2}^{1}\left(D\right)$ (in black) and $M_{2}\left(D+a_{2}\right)$ (in red) and graphical depiction of $I_{1}=\left[0,D_{1}\right),$ where *D*_1_ is the solution of $s_{2}^{0}\left(D\right)=s_{2}^{1}\left(D\right),$ and *I*_2_. (a): $I_{2}=\left[0,D_{2}\right)$ where *D*_2_ is the solution of $M_{2}\left(D+a_{2}\right)=s_{2}^{1}\left(D\right)$. (b) : $I_{2}=\left[0,D_{2}\right)$ where *D*_2_ is the solution of $M_{2}\left(D+a_{2}\right)=s_{2}^{0}\left(D\right)$. (c): *I*_2_ is empty. (d) : $I_{2}=\left(D_{2min},D_{2max}\right)$ where *D*_2*min*_ and *D*_2*max*_ are the solutions of $M_{2}\left(D+a_{2}\right)=s_{2}^{0}\left(D\right)$ and $M_{2}\left(D+a_{2}\right)=s_{2}^{1}\left(D\right),$ respectively. Cases (a)–(d) are obtained with the numerical parameter values listed in Tables 2 and 3. (For interpretation of the references to colour in this figure legend, the reader is referred to the web version of this article.)

The value ${\bar{s}}_{2}$ depends on *D*. However, to avoid cumbersome notation we will use the more precise form ${\bar{s}}_{2}\left(D\right)$ only if necessary.

We consider the function *F*_1_(*D*), which is the infimum of *ψ*(*s*_2_, *D*), with respect to the variable *s*_2_, see Fig. 2(b):

(30) $$\begin{array}{cc} F_{1}\left(D\right) & =\underset{s_{2}\in \left(s_{2}^{0}\left(D\right),s_{2}^{1}\left(D\right)\right)}{\inf}\psi \left(s_{2},D\right)=\psi \left({\bar{s}}_{2},D\right) \end{array}$$

We also define the functions *F*_2_(*D*) and *F*_3_(*D*):

(31) $$\begin{array}{cc} F_{2}\left(D\right) & =\psi \left(M_{2}\left(D+a_{2}\right),D\right) \end{array}$$

(32) $$\begin{array}{cc} F_{3}\left(D\right) & =\frac{\partial \psi }{\partial s_{2}}\left(M_{2}\left(D+a_{2}\right),D\right) \end{array}$$

The function *F*_1_(*D*) is defined for *D* ∈ *I*_1_ where:

(33) $$I_{1}=\left\{D\geq 0:s_{2}^{0}\left(D\right)<s_{2}^{1}\left(D\right)\right\}$$

The functions *F*_2_(*D*) and *F*_3_(*D*) are defined for *D* ∈ *I*_2_ where:

(34) $$I_{2}=\left\{D\in I_{1}:s_{2}^{0}\left(D\right)<M_{2}\left(D+a_{2}\right)<s_{2}^{1}\left(D\right)\right\}$$

For all for *D* ∈ *I*_2_, *F*_1_(*D*) ≤ *F*_2_(*D*). The equality $F_{1}\left(D\right)=F_{2}\left(D\right)$ holds if, and only if, $M_{2}\left(D+a_{2}\right)={\bar{s}}_{2}\left(D\right)$ that is, $\frac{\partial \psi }{\partial s_{2}}\left(M_{2}\left(D+a_{2}\right)\right)=0,$ that is if, and only if, $F_{3}\left(D\right)=0$. We define

$$I_{3}=\left\{D\in I_{2}:F_{3}\left(D\right)<0\right\}$$

For the Monod-type growth functions (Eq. 23), straightforward computations show that the functions *M*_0_, *M*_1_ and *M*_2_ are given respectively by:

For $y\in \left[0,\mu_{0}\left(+\infty ,s_{2}\right)=\frac{m_{0}s_{2}}{L_{0}+s_{2}}\right),$

$$M_{0}\left(y,s_{2}\right)=\frac{K_{0}y}{\frac{m_{0}s_{2}}{L_{0}+s_{2}}-y}$$

For $y\in \left[0,\mu_{1}\left(+\infty ,s_{2}\right)=\frac{m_{1}}{1+s_{2}/K_{i}}\right),$

$$M_{1}\left(y,s_{2}\right)=\frac{K_{1}y}{\frac{m_{1}}{1+s_{2}/K_{i}}-y}$$

For $y\in \left[0,\mu_{2}\left(+\infty \right)=m_{2}\right),$

$$M_{2}\left(y\right)=\frac{K_{2}y}{m_{2}-y}$$

Moreover, we have:

$$s_{2}^{0}\left(D\right)=\frac{L_{0}\left(D+a_{0}\right)}{m_{0}-D-a_{0}},\;s_{2}^{1}\left(D\right)=\frac{K_{i}\left(m_{1}-D-a_{1}\right)}{D+a_{1}}$$

$$\psi \left(s_{2},D\right)=\frac{K_{0}\left(D+a_{0}\right)}{m_{0}-D-a_{0}}\frac{L_{0}+s_{2}}{s_{2}-s_{2}^{0}\left(D\right)}+\frac{\frac{K_{1}\left(K_{i}+s_{2}\right)}{s_{2}^{1}\left(D\right)-s_{2}}+s_{2}}{1-\omega }$$

and

$$\frac{\partial^{2}\psi }{\partial s_{2}^{2}}\left(s_{2},D\right)=\frac{2K_{0}\left(D+a_{0}\right)}{m_{0}-D-a_{0}}\frac{L_{0}+s_{2}^{0}\left(D\right)}{{\left(s_{2}-s_{2}^{0}\left(D\right)\right)}^{3}}+\frac{2K_{1}\left(K_{i}+s_{2}^{1}\left(D\right)\right)}{\left(1-\omega \right){\left(s_{2}^{1}\left(D\right)-s_{2}\right)}^{3}}$$

Hence, $\frac{\partial^{2}\psi }{\partial s_{2}^{2}}>0$ for all $s_{2}\in \left(s_{2}^{0}\left(D\right),s_{2}^{1}\left(D\right)\right),$ so that the function *s*_2_↦*ψ*(*s*_2_, *D*) is convex and, thus, it satisfies assumption **H8**, see Fig. 2(b). The minimum ${\bar{s}}_{2}\left(D\right)$ is a solution of an algebraic equation of degree 4 in *s*_2_. Although mathematical software, such as *Maple*, cannot give solutions explicitly with respect to the parameters, ${\bar{s}}_{2}\left(D\right)$ could be obtained analytically since algebraic equations of degree 4 can theoretically be solved by quadratures. We do not try to obtain such an explicit formula. However, if the biological parameters are fixed, the function ${\bar{s}}_{2}\left(D\right)$ and, hence, $F_{1}\left(D\right)=\psi \left({\bar{s}}_{2}\left(D\right),D\right),$ can be obtained numerically. Since *M*_2_ and *ψ* are given explicitly the functions *F*_2_(*D*) and *F*_3_(*D*) are given explicitly with respect to the biological parameters in (Eq. 23). Since $D\mapsto s_{2}^{0}\left(D\right)$ is increasing and $D\mapsto s_{2}^{1}\left(D\right)$ is decreasing, and assuming $s_{2}^{0}\left(0\right)<s_{2}^{1}\left(0\right),$ the domain of definition *I*_1_ of *F*_1_(*D*) is an interval $I_{1}=\left[0,D_{1}\right),$ where *D*_1_ is the solution of $s_{2}^{0}\left(D\right)=s_{2}^{1}\left(D\right),$ see Fig. 3. For the domain of definition *I*_2_ of *F*_2_(*D*), several cases can be distinguished. *I*_2_ is an interval $I_{2}=\left[0,D_{2}\right),$ where *D*_2_ is the solution of $M_{2}\left(D+a_{2}\right)=s_{2}^{1}\left(D\right),$ see Fig. 3(a), or the solution of equation $M_{2}\left(D+a_{2}\right)=s_{2}^{0}\left(D\right),$ see Fig. 3(b). *I*_2_ is empty, see Fig. 3(c). *I*_2_ is an interval $I_{2}=\left(D_{2min},D_{2max}\right)$ where *D*_2*min*_ and *D*_2*max*_ are the solutions of $M_{2}\left(D+a_{2}\right)=s_{2}^{0}\left(D\right)$ and $M_{2}\left(D+a_{2}\right)=s_{2}^{1}\left(D\right),$ respectively, see Fig. 3(d).

## 5 Existence of steady-states

In this paper, as we are restricted only to local stability analysis, any reference to steady-state stability should be considered as local stability. A steady-state of ((14), (15), (16), (17), (18), (19)) is obtained by setting the right-hand sides equal to zero:

(35) $$\begin{array}{cc} \left[\mu_{0}\left(s_{0},s_{2}\right)-D-a_{0}\right]x_{0} & =0 \end{array}$$

(36) $$\begin{array}{cc} \left[\mu_{1}\left(s_{1},s_{2}\right)-D-a_{1}\right]x_{1} & =0 \end{array}$$

(37) $$\begin{array}{cc} \left[\mu_{2}\left(s_{2}\right)-D-a_{2}\right]x_{2} & =0 \end{array}$$

(38) $$\begin{array}{cc} D\left(s_{0}^{\text{in}}-s_{0}\right)-\mu_{0}\left(s_{0},s_{2}\right)x_{0} & =0 \end{array}$$

(39) $$\begin{array}{cc} -Ds_{1}+\mu_{0}\left(s_{0},s_{2}\right)x_{0}-\mu_{1}\left(s_{1},s_{2}\right)x_{1} & =0 \end{array}$$

(40) $$\begin{array}{cc} -Ds_{2}+\mu_{1}\left(s_{1},s_{2}\right)x_{1}-\omega \mu_{0}\left(s_{0},s_{2}\right)x_{0}-\mu_{2}\left(s_{2}\right)x_{2} & =0 \end{array}$$

A steady-state exists (or is said to be ‘meaningful’) if, and only if, all its components are non-negative.

Lemma 2 *The only steady-state of* (Eqs. 14*–*19*), for which*$x_{0}=0$ *or*$x_{1}=0,$ *is the washout steady-state*$\text{SS}1=(x_{0},x_{1},x_{2},s_{0},s_{1},s_{2}),$ *where*$x_{0}=0,$$x_{1}=0,$$x_{2}=0,$$s_{0}=s_{0}^{in},$$s_{1}=0$ *and*$s_{2}=0$*. This steady-state always exists and is always locally stable.*

Besides the steady-state SS1, the system can have at most two other type of steady-states.

• SS2: *x*_0_ > 0, *x*_1_ > 0 and $x_{2}=0,$ where species *x*_2_ is washed out, while species *x*_0_ and and *x*_1_ exist. We show below that, generally, the system can have two steady-states SS2^♭^ and SS2^♯^.
• SS3: *x*_0_ > 0, *x*_1_ > 0, and *x*_2_ > 0, where all populations are maintained. We show below that the steady-state SS3 is unique if it exists.

We can state now the necessary and sufficient conditions of existence of SS2 and SS3.

Lemma 3 *If ω* ≥ 1 *then SS2 does not exist. If ω* < 1 *then SS2 exists if, and only if,*$s_{0}^{\text{in}}\geq F_{1}\left(D\right)$*. Therefore, a necessary condition for the existence of SS2 is that D* ∈ *I*_1_, *where I*_1_ *is defined by (Eq.* 33*). If*$s_{0}^{\text{in}}\geq F_{1}\left(D\right)$ *then each solution s*_2_ *of equation*

(41) $$\psi \left(s_{2}\right)=s_{0}^{\text{in}},\;s_{2}\in \left(s_{2}^{0},s_{2}^{1}\right)$$

*gives a steady-state*$\text{SS}2=(x_{0},x_{1},x_{2},s_{0},s_{1},s_{2})$ *where*

(42) $$\begin{array}{cc} & s_{0}=M_{0}\left(D+a_{0},s_{2}\right),\;s_{1}=M_{1}\left(D+a_{1},s_{2}\right)\\ & x_{0}=\frac{D}{D+a_{0}}\left(s_{0}^{\text{in}}-s_{0}\right),\;x_{1}=\frac{D}{D+a_{1}}\left(s_{0}^{\text{in}}-s_{0}-s_{1}\right),\;x_{2}=0 \end{array}$$

Lemma 4 *If ω* ≥ 1 *then SS3 does not exist. If ω* < 1 *then SS3 exists if, and only if,*$s_{0}^{\text{in}}>F_{2}\left(D\right)$*. Therefore, a necessary condition of existence of SS3 is that D* ∈ *I*_2_, *where I*_2_ *is defined by (Eq.* 34*). If*$s_{0}^{\text{in}}>F_{2}\left(D\right)$ *then the steady-state*$\text{SS}3=\left(x_{0},x_{1},x_{2},s_{0},s_{1},s_{2}\right)$ *is given by*

(43) $$\begin{array}{ccc} & & s_{0}=M_{0}\left(D+a_{0},M_{2}\left(D+a_{2}\right)\right),\;s_{1}=M_{1}\left(D+a_{1},M_{2}\left(D+a_{2}\right)\right),\\ & & s_{2}=M_{2}\left(D+a_{2}\right) \end{array}$$

*called the break-even concentrations, and*

(44) $$\begin{array}{ccc} & & x_{0}=\frac{D}{D+a_{0}}\left(s_{0}^{\text{in}}-s_{0}\right),\;x_{1}=\frac{D}{D+a_{1}}\left(s_{0}^{\text{in}}-s_{0}-s_{1}\right),\\ & & x_{2}=\frac{D}{D+a_{2}}\left(\left(1-\omega \right)\left(s_{0}^{\text{in}}-s_{0}\right)-s_{1}-s_{2}\right) \end{array}$$

Remark 1 If $s_{0}^{\text{in}}>F_{1}\left(D\right)$ then (Eq. 41) has exactly two solutions denoted by $s_{2}^{♭}$ and $s_{2}^{♯}$ and such that, see Fig. 2(c),

$$s_{2}^{0}<s_{2}^{♭}<{\bar{s}}_{2}<s_{2}^{♯}<s_{2}^{1}$$

If $s_{0}^{\text{in}}=F_{1}\left(D\right)$ then $s_{2}^{0}<s_{2}^{♭}={\bar{s}}_{2}=s_{2}^{♯}<s_{2}^{1}$.

To these solutions, $s_{2}^{♭}$ and $s_{2}^{♯},$ correspond two steady-states of SS2, which are denoted by SS2^♭^ and SS2^♯^. These steady-states coalesce when $s_{0}^{\text{in}}=F_{1}\left(D\right)$.

Since *F*_1_(*D*) ≤ *F*_2_(*D*), the condition $s_{0}^{\text{in}}>F_{2}\left(D\right)$ for the existence of the positive steady-state SS3 implies that the condition $s_{0}^{\text{in}}>F_{1}\left(D\right)$ for the existence of the two steady-states SS2^♭^ and SS2^♯^ is satisfied. Therefore, if SS3 exists then SS2^♭^ and SS2^♯^ exist and are distinct. If $s_{0}^{\text{in}}=F_{2}\left(D\right)$ then SS3 coalesces with SS2^♭^ if *F*_3_(*D*) < 0, and with SS2^♯^ if *F*_3_(*D*) > 0, respectively.

Remark 2 Using (Eq. 20), the conditions $s_{0}^{\text{in}}>F_{1}\left(D\right)$ and $s_{0}^{\text{in}}>F_{2}\left(D\right)$ of existence of the steady-state SS2 and SS3, respectively are equivalent to the conditions

$$S_{\text{ch},\text{in}}>\frac{F_{1}\left(D\right)}{Y_{3}Y_{4}}\;\text{and}\;S_{\text{ch},\text{in}}>\frac{F_{2}\left(D\right)}{Y_{3}Y_{4}}$$

respectively expressed with respect to the inflowing concentration *S*_ch, in_. Moreover, for the nominal parameter values given in Table 1, we have *ω* ≈ 0.53. Therefore, the steady-states SS2 and SS3 exist if the conditions on the operating parameters stated in Lemmas 3 and 4, respectively are satisfied, see Table 7.

## 6 Operating diagram

The *Operating diagrams* show how the system behaves when we vary the two control parameters *S*_ch, in_ and *D* (i.e., substrate inflow rate and medium flow-rate per culture volume) in ((1), (2), (3), (4), (5), (6)). This conventional bifurcation plot is used to visualise the existence and stability of steady-states with respect to these operating parameters, as they are the parameters most easily manipulated in a chemostat by which the system under scrutiny can be examined.

**Fig. 4:**
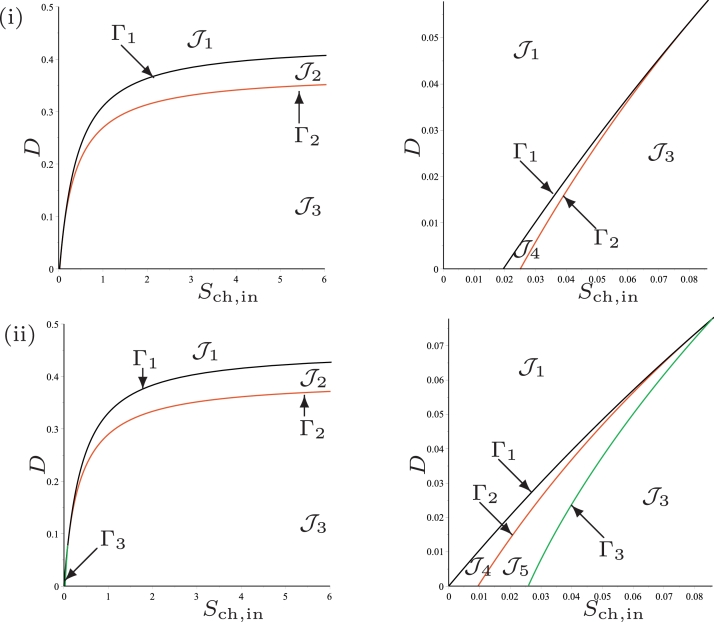
The curves *Γ*_1_ (black), *Γ*_2_ (red) and *Γ*_3_ (green) for case (a). (i) : regions of steady-state existence, with maintenance. On the right, a magnification for $0<D<D_{3}=0.058$ showing the region $J_{4}$. (ii) : regions of steady-state existence and their stability, without maintenance. On the right, a magnification for $0<D<D_{3}=0.078$ showing the regions $J_{4}$ and $J_{5}$. (For interpretation of the references to colour in this figure legend, the reader is referred to the web version of this article.)

According to Remark 2, the curve *Γ*_1_ of equation

(45) $$S_{\text{ch},\text{in}}=\frac{1}{Y_{3}Y_{4}}F_{1}\left(D\right)$$

is the border to which SS2 exists, and the curve *Γ*_2_ of equation

(46) $$S_{\text{ch},\text{in}}=\frac{1}{Y_{3}Y_{4}}F_{2}\left(D\right)$$

is the border to which SS3 exists, see Fig. 4. If we want to plot the operating diagram we must fix the values of the biological parameters. In the remainder of the Section we plot the operating diagrams corresponding to cases (a)–(d) depicted in Fig. 3.

### 6.1 Operating diagram: case (a)

This case corresponds to the parameter values used by [24]. We have seen in Table 2 that the curves *Γ*_1_ and *Γ*_2_ are defined for *D* < *D*_1_ and *D* < *D*_2_, respectively and that they are tangent for $D=D_{3},$ where $D_{1}=0.432,$$D_{2}=0.373$ and $D_{3}=0.058$. Therefore, they separate the operating plane (*S*_ch, in_, *D*) into four regions, as shown in Fig. 4(i), labelled $J_{1},$$J_{2}$ and $J_{3}$ and $J_{4}$.

**Table 2:** Parameter values of the maintenance terms *a_i_*, i=0,1,2, for cases (a), (b) and (c) of Fig. 3. Unspecified parameter values are as in Table 1. The table gives the values of *D*_1_, *D*_2_ and *D*_3_ where $I_{1}=\left[0,D_{1}\right),$$I_{2}=\left[0,D_{2}\right)$ and $I_{3}=\left[0,D_{3}\right)$.

	$K_{S,H_{2},c}$	*a_i_*	*D*_1_	*D*_2_	*D*_3_
(a)	$1.0\times 10^{-6}$	0.02	0.432	0.373	0.058
		0	0.452	0.393	0.078
(b)	$4.0\times 10^{-6}$	0.02	0.329	0.236	$I_{3}=I_{2}$
		0	0.349	0.256	$I_{3}=I_{2}$
(c)	$7.0\times 10^{-6}$	0.02	0.287	$I_{2}=\emptyset$	
		0	0.303	$I_{2}=\emptyset$	

The results are summarised in Table 4, which shows the existence of the steady-states SS1, SS2 and SS3 in the regions of the operating diagram in Fig. 4(i).

### 6.2 Operating diagram: case (b)

**Fig. 5:**
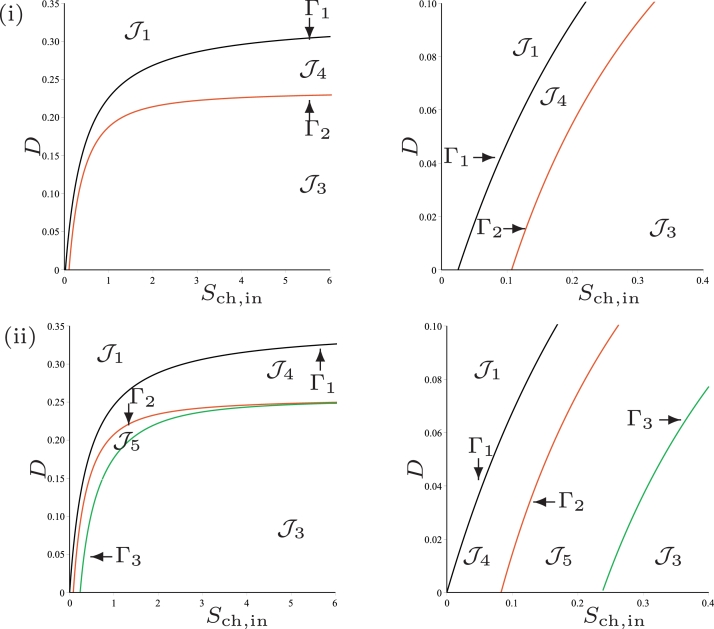
The curves *Γ*_1_ (black), *Γ*_2_ (red) and *Γ*_3_ (green) for case (b). (i) : regions of steady-state existence, with maintenance. (ii) : regions of steady-state existence and their stability, without maintenance. On the right, a magnification for 0 < *D* < 0.1. (For interpretation of the references to colour in this figure legend, the reader is referred to the web version of this article.)

This case corresponds to the parameter values used by [24], except that $K_{S,H_{2},c}$ is changed from $1.0\times 10^{-6}$ to $4.0\times 10^{-6}$. We have seen in Table 2 that the curves *Γ*_1_ and *Γ*_2_ are defined for *D* < *D*_1_ and *D* < *D*_2_, respectively and *F*_1_(*D*) < *F*_2_(*D*) for all *D* < *D*_2_, where $D_{1}=0.329$ and $D_{2}=0.236$. Therefore, they separate the operating plane (*S*_ch, in_, *D*) in three regions, as shown in Fig. 5(i), labelled $J_{1},$$J_{3}$ and $J_{4}$.

The results are summarised in Table 5, which shows the existence of the steady-states SS1, SS2 and SS3 in the regions of the operating diagram in Fig. 5(i). Note that the region $J_{2}$ has disappeared.

### 6.3 Operating diagram: case (c)

**Fig. 6:**
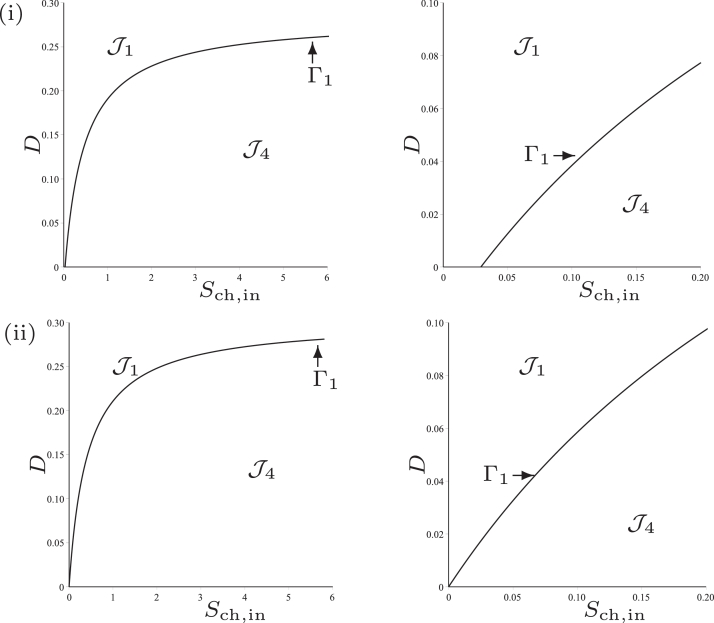
The curve *Γ*_1_ for case (c). (i) : regions of steady-state existence, with maintenance. (ii) : regions of steady-state existence and their stability, without maintenance. On the right, a magnification for 0 < *D* < 0.1.

This case corresponds to the parameter values used by [24], except that $K_{S,H_{2},c}$ is changed from $1.0\times 10^{-6}$ to $7.0\times 10^{-6}$. We have seen in Table 2 that the curve *Γ*_1_ is defined for $D<D_{1}=0.287$ and that *I*_2_ is empty so that SS3 does not exist. Therefore, *Γ*_1_ separates the operating plane (*S*_ch, in_, *D*) in two regions, as shown in Fig. 6(i), labelled $J_{1}$ and $J_{4}$.

The results are summarised in Table 6, which shows the existence of the steady-states SS1 and SS2 in the regions of the operating diagram in Fig. 6(i). Note that the region $J_{3}$ of existence of SS3 has disappeared.

### 6.4 Operating diagram: case (d)

**Fig. 7:**
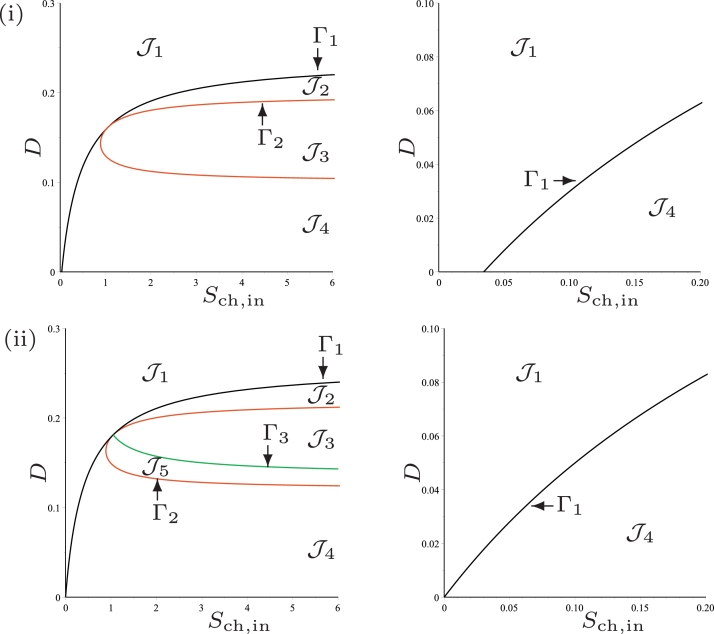
The curves *Γ*_1_ (black), *Γ*_2_ (red) and *Γ*_3_ (green) for case (d). (i) : regions of steady-state existence, with maintenance. (ii) : regions of steady-state existence and their stability, without maintenance. On the right, a magnification for 0 < *D* < 0.1. (For interpretation of the references to colour in this figure legend, the reader is referred to the web version of this article.)

We end this discussion on the role of kinetic parameters by the presentation of this case, which presents a new behaviour that did not occur in the preceding cases: there exists *D*_2*min*_ such that for *D* < *D*_2*min*_ the system cannot have a positive steady-state SS3. This case corresponds to the parameter values used by [24], except that three of them are changed as indicated in Table 3. This table shows that the curves *Γ*_1_ and *Γ*_2_ are defined for *D* < *D*_1_ and *D*_2*min*_ < *D* < *D*_2*max*_ and that they are tangent for $D=D_{3},$ where $D_{1}=0.238,$$D_{2min}=0.101,$$D_{2max}=0.198$ and $D_{3}=0.161$. Therefore, *Γ*_1_ and *Γ*_2_ separate the operating plane (*S*_ch, in_, *D*) in four regions, as shown in Fig. 7(i), labelled $J_{1},$$J_{2},$$J_{3}$ and $J_{4}$. The results are summarised in Table 4, which shows the existence of the steady-states SS1, SS2 and SS3 in the regions of the operating diagram in Fig. 7(i).

**Table 3:** Parameter values of the maintenance terms *a_i_*, i=0,1,2, for case (d) of Fig. 3: $K_{S,H_{2},c}=1.2\times 10^{-5},$$K_{S,H_{2}}=0.5\times 10^{-5}$ and $k_{m,H_{2}}=5$. Unspecified parameter values are as in Table 1. The table gives the values of *D*_1_, *D*_2*min*_, *D*_2*max*_ and *D*_3_ where $I_{1}=\left[0,D_{1}\right),$$I_{2}=\left(D_{2min},D_{2max}\right)$ and $I_{3}=\left(D_{2min},D_{3}\right)$.

	*a_i_*	*D*_1_	*D*_2*min*_	*D*_2*max*_	*D*_3_
(d)	0.02	0.238	0.101	0.198	0.161
	0	0.258	0.121	0.218	0.181

**Table 4:** Existence of steady-states in the regions of the operating diagrams of Fig. 4(i) and Fig. 7(i).

Region	Steady-states
$J_{1}$	SS1
$J_{2}\cup J_{4}$	SS1, SS2^♭^, SS2^♯^
$J_{3}$	SS1, SS2^♭^, SS2^♯^, SS3

**Table 5:** Existence of steady-states in the regions of the operating diagram of Fig 5(i).

Region	Steady-states
$J_{1}$	SS1
$J_{4}$	SS1, SS2^♭^, SS2^♯^
$J_{3}$	SS1, SS2^♭^, SS2^♯^, SS3

**Table 6:** Existence of steady-states in the regions of the operating diagram of Fig 6(i).

Region	Steady-states
$J_{1}$	SS1
$J_{4}$	SS1, SS2^♭^, SS2^♯^

**Table 7:** Existence (with or without maintenance) and stability (without maintenance) of steady-states. The conditions for existence and stability are satisfied given that the inequalities are observed.

	Existence	Stability
SS1	Always exists	Always stable
SS2^♭^	$s_{0}^{\text{in}}>F_{1}\left(D\right)$	Always unstable
SS2^♯^	$s_{0}^{\text{in}}>F_{1}\left(D\right)$	*F*_3_(*D*) > 0 and $s_{0}^{in}<F_{2}\left(D\right)$
SS3	$s_{0}^{\text{in}}>F_{2}\left(D\right)$	*F*_3_(*D*) ≥ 0 or
		*F*_3_(*D*) < 0 and $F_{4}\left(D,s_{0}^{\text{in}}\right)>0$

## 7 Local stability without maintenance

We know that SS1 is always stable. However, the analytical study of the stability of SS2 and SS3 is very difficult because the conditions for Routh-Hurwitz in the 6-dimensional case are intractable. For this reason we will consider in this section the question of the stability only for the case without maintenance ($a_{0}=a_{1}=a_{2}=0$), since the system reduces to a three-dimensional system. The general case will be considered only numerically in Section 8. When maintenance is not considered in the model, the steady-states SS1, SS2 and SS3 are given by:

1. $\text{SS}1=(0,0,0,s_{0}^{\text{in}},0,0)$
2. $\text{SS}2=(x_{0},x_{1},0,s_{0},s_{1},s_{2})$ where *s*_2_ a solution of equation
  $$s_{0}^{\text{in}}=\psi \left(s_{2}\right)=M_{0}\left(D,s_{2}\right)+\frac{M_{1}\left(D,s_{2}\right)+s_{2}}{1-\omega }$$
  and
  (47) $$s_{0}=M_{0}\left(D,s_{2}\right),\;\;\;s_{1}=M_{1}\left(D,s_{2}\right)\;x_{0}=s_{0}^{\text{in}}-s_{0},\;\;\;x_{1}=s_{0}^{\text{in}}-s_{0}-s_{1}$$
3. $\text{SS}3=(x_{0},x_{1},x_{2},s_{0},s_{1},s_{2})$ where
  (48) $$\begin{array}{cc} s_{2} & =M_{2}\left(D\right),\;s_{0}=M_{0}\left(D,s_{2}\right),\;s_{1}=M_{1}\left(D,s_{2}\right)\\ x_{0} & =s_{0}^{\text{in}}-s_{0},\\ x_{1} & =s_{0}^{\text{in}}-s_{0}-s_{1}{,}_{q}uadx_{2}=\left(1-\omega \right)\left(s_{0}^{\text{in}}-s_{0}\right)-s_{1}-s_{2} \end{array}$$

Proposition 2 *Let*$\text{SS}2=(x_{0},x_{1},0,s_{0},s_{1},s_{2})$ *be a steady-state. Then SS2 is stable if, and only if, μ*_2_(*s*_2_) < *D and*$\frac{\partial \psi }{\partial s_{2}}>0$*.*

Therefore, SS2^♭^ is always unstable and SS2^♯^ is stable if, and only if, *μ*_2_(*s*_2_) < *D*. This last condition is equivalent to $M_{2}\left(D\right)>s_{2}^{♯},$ which implies that *F*_3_(*D*) > 0. Hence, if SS3 exists then SS2^♯^ is necessarily unstable. Therefore, SS2^♯^ is stable if, and only if, *F*_3_(*D*) > 0 and SS3 does not exist.

Proposition 3 *Let*$\text{SS}3=(x_{0},x_{1},x_{2},s_{0},s_{1},s_{2})$ *be a steady-state. If F*_3_(*D*) ≥ 0 *then SS3 is stable as long as it exists. If F*_3_(*D*) < 0 *then SS3 can be unstable. The instability of SS3 occurs inparticular when s*_2_ *is sufficiently close to*$s_{2}^{♭},$ *i.e. when SS3 is sufficiently close to* SS2^♭^*.*

The condition *F*_3_(*D*) ≥ 0 is equivalent to $\frac{\partial \psi }{\partial s_{2}}\left(M_{2}\left(D\right)\right)\geq 0,$ namely $s_{2}=M_{2}\left(D\right)\in \left[{\bar{s}}_{2},s_{2}^{♯}\right)$. If $\frac{\partial \psi }{\partial s_{2}}<0,$ viz. $s_{2}\in \left(s_{2}^{♭},{\bar{s}}_{2}\right),$ then SS3 can be unstable.

When *D* is such that *F*_3_(*D*) < 0, the determination of the boundary between the regions of stability and instability of SS3 needs to examine the Routh-Hurwitz condition of stability for SS3. For this purpose we define the following functions. Let $\text{SS}3=(x_{0},x_{1},x_{2},s_{0},s_{1},s_{2})$ be a steady-state and

$$E=\frac{\partial \mu_{0}}{\partial s_{0}},\;F=\frac{\partial \mu_{0}}{\partial s_{2}},\;G=\frac{\partial \mu_{1}}{\partial s_{1}},\;H=-\frac{\partial \mu_{1}}{\partial s_{2}},\;I=\frac{d\mu_{2}}{ds_{2}}$$

evaluated at the steady-state SS3 defined by (48), i.e. for

$$s_{2}=M_{2}\left(D\right),\;s_{0}=M_{0}\left(D,s_{2}\right),\;s_{1}=M_{1}\left(D,s_{2}\right)$$

For *D* ∈ *I*_3_ and $s_{0}^{\text{in}}>F_{2}\left(D\right),$ we define:

(49) $$\begin{array}{ccc} F_{4}\left(D,s_{0}^{\text{in}}\right) & = & \left(EIx_{0}x_{2}+\left[E(G+H)-(1-\omega )FG\right]x_{0}x_{1}\right)f_{2}\\ & & +\left(Ix_{2}+\left(G+H\right)x_{1}+\omega Fx_{0}\right)GIx_{1}x_{2} \end{array}$$

where $f_{2}=Ix_{2}+\left(G+H\right)x_{1}+\left(E+\omega F\right)x_{0}$. Notice that to compute $F_{4}\left(D,s_{0}^{\text{in}}\right)$ we must replace *x*_0_, *x*_1_, *x*_2_, *s*_0_, *s*_1_ and *s*_2_ by their values at SS3, given by (48). Hence, this function depends on the operating parameters *D* and $s_{0}^{\text{in}}$. For each fixed *D* ∈ *I*_3_, $F_{4}\left(D,s_{0}^{\text{in}}\right)$ is polynomial in $s_{0}^{\text{in}}$ of degree 3 and tends to +∞ when $s_{0}^{\text{in}}$ tends to +∞. Therefore, it is necessarily positive for large enough $s_{0}^{\text{in}}$. The values of the operating parameters *D* and $s_{0}^{\text{in}}$ for which $F_{4}\left(D,s_{0}^{\text{in}}\right)$ is positive correspond to the stability of SS3 as shown in the following proposition.

Proposition 4 *Let*$\text{SS}3=(x_{0},x_{1},x_{2},s_{0},s_{1},s_{2})$ *be a steady-state. If F*_3_(*D*) < 0 *then SS3 is stable if, and only if,*$F_{4}\left(D,s_{0}^{\text{in}}\right)>0$*.*

The results on the existence of steady-states (with or without maintenance) of Lemmas 2–4, and their stability (without maintenance) of Props 2–4, are summarised in Table 7.

### 7.1 Operating diagram: case (a)

This case corresponds to the parameter values used by [24] but without maintenance. We see from Table 2 that the curves *Γ*_1_ and *Γ*_2_ of the operating diagram, given by (Eq. 45) and (Eq. 46), respectively are defined now for *D* < *D*_1_ and *D* < *D*_2_, respectively and that they are tangent for $D=D_{3},$ where $D_{1}=0.452,$$D_{2}=0.393$ and $D_{3}=0.078$. Beside these curves, we plot also on the operating diagram of Fig. 4(ii), the curve *Γ*_3_ of equation

(50) $$F_{4}\left(D,Y_{3}Y_{4}S_{\text{ch},\text{in}}\right)=0$$

According to Prop. 4, this curve is defined for $D<D_{3}=0.078$ and it separates the region of existence of SS3 into two subregions labelled $J_{3}$ and $J_{5},$ such that SS3 is stable in $J_{3}$ and unstable in $J_{5}$. The other regions $J_{1},$$J_{2}$ and $J_{4}$ are defined as in the previous section. The operating diagram is shown Fig. 4(ii). It looks very similar to Fig. 4(i), except near the origin, as it is indicated in the magnification for $0<D<D_{3}=0.078$. From Table 7, we deduce the following result

Proposition 5 Table 8 *shows the existence and stability of the steady-states SS1, SS2 and SS3 in the regions of the operating diagram in* Fig. 4*(ii).*

**Table 8:** Existence and stability of steady-states in the regions of the operating diagrams of Fig. 4(ii) and Fig. 7(ii)., where S and U indicate stable and unstable steady-states, respectively.

Region	SS1	SS2^♭^	SS2^♯^	SS3
$J_{1}$	S			
$J_{2}$	S	U	S	
$J_{3}$	S	U	U	S
$J_{4}$	S	U	U	
$J_{5}$	S	U	U	U

### 7.2 Operating diagram: case (b)

We see from Table 2 that the curves *Γ*_1_ and *Γ*_2_ are defined now for $D<D_{1}=0.349$ and $D<D_{2}=0.256,$ respectively and that *F*_1_(*D*) < *F*_2_(*D*) for all *D*. Beside these curves, we plot also on the operating diagram of Fig. 5(ii), the curve *Γ*_3_ of equation (Eq. 50) which separates the region of existence of SS3 into two subregions labelled $J_{3}$ and $J_{5},$ such that SS3 is stable in $J_{3}$ and unstable in $J_{5}$. Therefore, the curves *Γ*_1_, *Γ*_2_ and *Γ*_3_ separate the operating plane (*S*_ch, in_, *D*) into four regions, as shown in Fig. 5(ii), labelled $J_{1},$$J_{3},$$J_{4}$ and $J_{5}$.

Proposition 6 Table 9 *shows the existence and stability of the steady-states SS1, SS2 and SS3 in the regions of the operating diagram in* Fig. 5*(ii)*.

**Table 9:** Existence and stability of steady-states in the regions of the operating diagram of Fig. 5(ii).

Region	SS1	SS2^♭^	SS2^♯^	SS3
$J_{1}$	S			
$J_{3}$	S	U	U	S
$J_{4}$	S	U	U	
$J_{5}$	S	U	U	U

### 7.3 Operating diagram: case (c)

We see from Table 2 that *Γ*_1_ is defined for $D<D_{1}=0.303$ and that *I*_2_ is empty so that SS3 does not exist. Therefore, *Γ*_1_ separates the operating plane (*S*_ch, in_, *D*) into two regions, as shown in Fig. 6(ii), labelled $J_{1}$ and $J_{4}$.

Proposition 7 Table 10 *shows the existence and stability of the steady-states SS1, SS2 and SS3 in the regions of the operating diagram in* Fig. 6*(ii).*

**Table 10:** Existence and stability of steady-states in the regions of the operating diagram of Fig. 6(ii).

Region	SS1	SS2^♭^	SS2^♯^	SS3
$J_{1}$	S			
$J_{4}$	S	U	U

### 7.4 Operating diagram: case (d)

We see in Table 3 that the curves *Γ*_1_ and *Γ*_2_ are defined for *D* < *D*_1_ and *D*_2*min*_ < *D* < *D*_2*max*_ and that they are tangent for $D=D_{3},$ where $D_{1}=0.258$ and $D_{2min}=0.121,$$D_{2max}=0.218$ and $D_{3}=0.181$. Beside these curves, we plot also on the operating diagram of Fig. 7(ii), the curve *Γ*_3_ defined by (Eq. 50), which separates the region of existence of SS3 into two subregions labelled $J_{3}$ and $J_{5},$ such that SS3 is stable in $J_{3}$ and unstable in $J_{5}$. Therefore, the curves *Γ*_1_, *Γ*_2_ and *Γ*_3_ separate the operating plane (*S*_ch, in_, *D*) into five regions, as shown in Fig. 7(ii), labelled $J_{1},$$J_{2},$$J_{3},$$J_{4}$ and $J_{5}$.

Proposition 8 Table 8 *shows the existence and stability of the steady-states SS1, SS2 and SS3 in the regions of the operating diagram in* Fig. 7*(ii).*

## 8 Numerical analysis to illustrate the analytical results

The aim of this section is to study numerically (the method is explained in Appendix A) the existence and stability of the steady-states SS2 and SS3. We obtain numerically the operating diagrams that were described in Sections 5 and 7. The results in this section confirm the results on existence of the steady-states obtained in Section 5 in the cases with or without maintenance and the results of stability obtained in Section 7 in the case without maintenance. These results permit also to extend the analytical results and elucidate the problem of the local stability of SS2 and SS3, which was left open in Section 7.

### 8.1 Operating diagram: case (a)

**Fig. 8:**
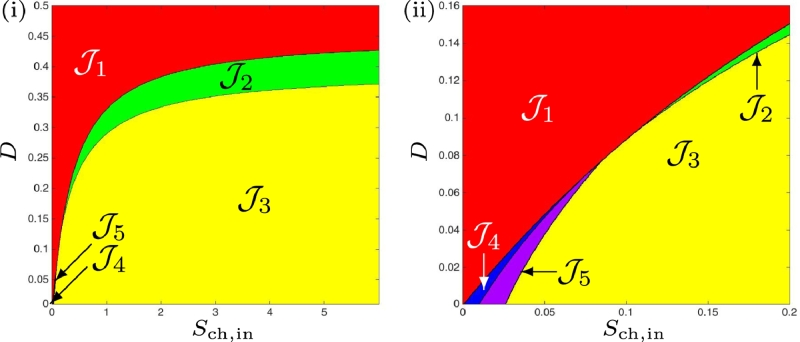
Numerical analysis for the existence and stability of steady-states for case (a), without maintenance. On the right, a magnification for 0 < *D* < 0.16.

**Fig. 9:**
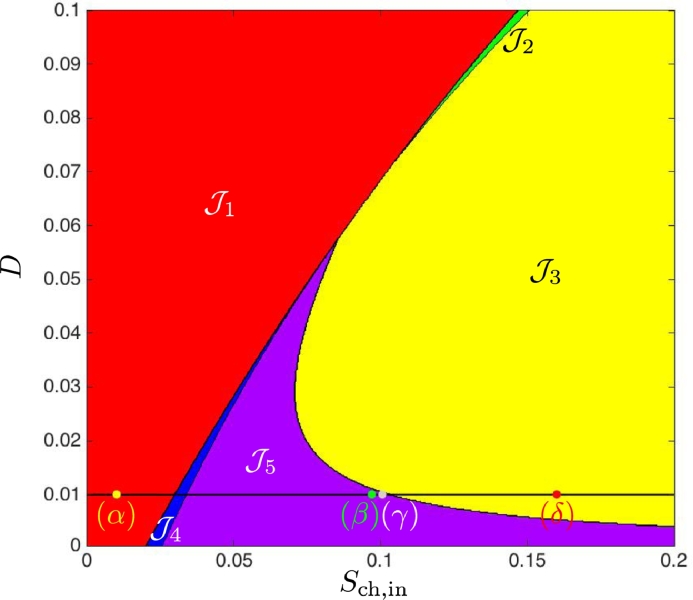
Numerical analysis for the existence and stability of steady-states for case (a), with maintenance. This is a magnification for 0 < *D* < 0.1, showing the presence and extent of region $J_{5}$ undetectable by the analytical method. The coordinates labelled (α)−(δ) are subsequently used to simulate the system dynamics, as shown in the proceeding Fig. 10.

**Fig. 10:**
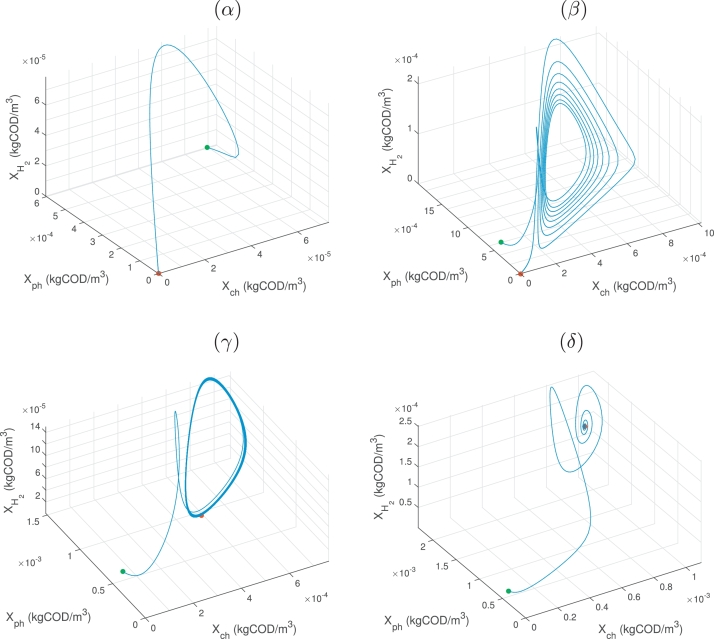
Three-dimensional phase plane diagrams of the biomass dynamics for t=10000d, showing initial (green dot) and final (red dot) conditions for a dilution rate, $D=0.01\;d^{-1}$ and chlorophenol input, *S*_ch, *in*_ (*kgCOD*/*m*^3^), of (*α*): $S_{\text{ch},in}=0.01$ - the system converges to SS1, (*β*): $S_{\text{ch},in}=0.097$ - the system enters a periodic orbit of increasing amplitude, ultimately converging to SS1, (*γ*): $S_{\text{ch},in}=0.10052$ - the system is close to a stable limit cycle, (*δ*): $S_{\text{ch},in}=0.16$ - the system undergoes damped oscillations and converges to SS3. (For interpretation of the references to colour in this figure legend, the reader is referred to the web version of this article.)

We endeavoured to find numerically the operating conditions under which SS3 is unstable, previously unreported by [24]. Given that we have determined analytically in Proposition 3 that when SS3 is close to SS2^♭^ it becomes unstable, we performed numerical simulations with the parameters defined in Table 1 over an operating region similar to that shown in Fig. 2 from [24] whilst also satisfying our conditions. In Fig. 8 we show the case when maintenance is excluded. When magnified, we observe more clearly that region $J_{5}$ does exist for the conditions described above, and also note that the region $J_{4}$ occurs in a small area between $J_{1}$ and $J_{5},$ which corresponds to the results shown in Fig. 4(ii), and is in agreement with Proposition 5. In Fig. 9 we confirm that region $J_{5}$ does exist for the conditions described above, when maintenance is included, but could not be determined analytically, the curve *Γ*_3_ is absent in Fig. 4(i). Furthermore, we demonstrate that there is evidence of Hopf bifurcation, which occurs along the boundary of *F*_3_(*D*) for values of *D* < *D*_3_ by selecting values of *S*_ch, in_ (indicated by (α)−(δ) in Fig. 9) at a fixed dilution rate of $0.01\;d^{-1},$ and running dynamic simulations for 10000 *d*. The three-dimensional phase plots, with the axes representing biomass concentrations, are shown in Fig. 10, and show that as *S*_ch, in_ approaches $J_{3}$ from $J_{5},$ emergent periodic orbits are shown to diminish to a stable limit cycle at the boundary (see  Appendix B for details). Subsequently, increasing *S*_ch, in_ to $J_{3}$ results in the orbit reducing to a fixed point equilibrium at SS3.

### 8.2 Operating diagram: case (b)

**Fig. 11:**
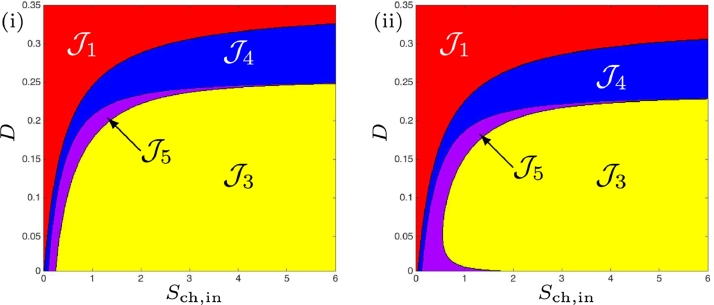
Numerical analysis for the existence and stability of steady-states for case (b). (i) : without maintenance. (ii) : with maintenance.

Whilst the numerical parameters chosen for this work are taken from the original study [24], there somewhat arbitrary nature leaves room to explore the impact of the parameters on the existence and stability of the steady-states. Case (b), discussed in Sections 6.2 and 7.2, involves a small increase to the half-saturation constant (or inverse of substrate affinity), $K_{S,H_{2},c},$ of the chlorophenol degrader on hydrogen. Following the same approach as with the preceding case, we confirm in Fig. 11(i) the Proposition 6 in the scenario without maintenance. Furthermore, the extension of this proposition with maintenance included, corresponding to the existence and stability of all three steady-states given in Table 9, is shown in Fig 11(ii). It shows the region $J_{5}$ that cannot be obtained analytically (cf. Fig. 5(i)). In both cases, region $J_{2}$ has disappeared, as observed analytically. Additionally, the ideal region $J_{3},$ where all organisms are present and stable, diminishes.

### 8.3 Operating diagram: case (c)

**Fig. 12:**
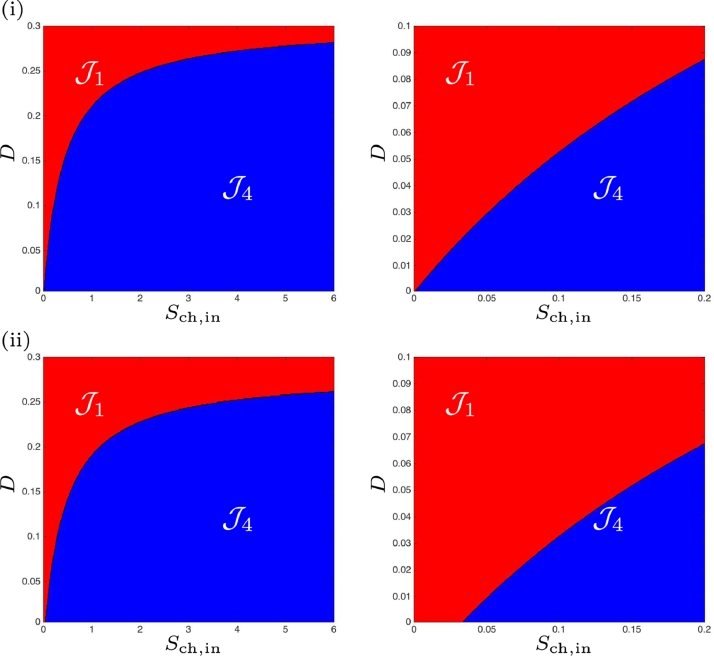
Numerical analysis for the existence and stability of steady-states for case (c). (i) : without maintenance. (ii) : with maintenance. On the right, a magnification for 0 < *D* < 0.1.

Here, $K_{S,H_{2},c},$ was further increased and confirm the Proposition 7, where the function SS3 never exist and SS2 never stable for the case without maintenance. The extension of this proposition to the case with maintenance, shown in Table 10, produce similar results as shown in the comparison of Fig. 12(i) and (ii).

### 8.4 Operating diagram: case (d)

**Fig. 13:**
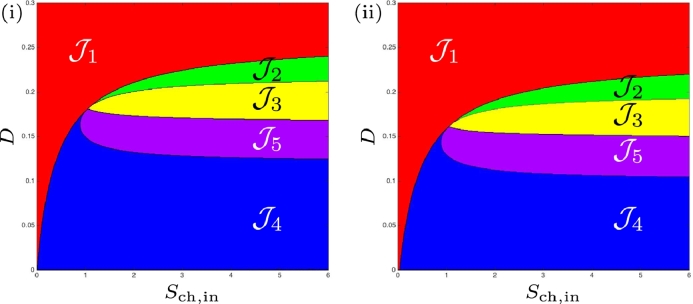
Numerical analysis for the existence and stability of steady-states for case (d). (i) : without maintenance. (ii) : with maintenance.

With the final investigated scenario, where $k_{m,H_{2}}<k_{m,\text{ch}}$ and $K_{S,H_{2}}<K_{S,H_{2},c},$ we observe once again the presence of all operating regions, $J_{1}-J_{5},$ without and with maintenance, as shown in Fig. 13. It can be seen that regions $J_{4}$ and $J_{5}$ increase at low dilution rates across a much larger range of *S*_ch, in_ than in the default case (a), and the desired condition (stable SS3) is restricted to a much narrower set of *D*.

As with the previous cases, the numerical analysis for case (d) confirms the Proposition 8 without maintenance and its extension to the case with maintenance, indicated in Table 8.

## 9 The role of kinetic parameters

Finally, we give brief consideration to the characterisation of the four cases discussed in the preceding sections. The main difference between cases (a) or (b) and cases (c) or (d) is that, for small values of *D*, the coexistence steady-state SS3 can exist for cases (a) and (b), but cannot exist for cases (c) or (d). The cases (a) or (b) occur if and only if $s_{2}^{0}\left(0\right)<M_{2}\left(0\right)$ holds or $s_{2}^{0}\left(0\right)=M_{2}\left(0\right)$ and $\frac{ds_{2}^{0}}{dD}\left(0\right)<\frac{dM_{2}}{dD}\left(0\right)$ hold, viz.

(51) $$\begin{array}{c} \frac{L_{0}a_{0}}{m_{0}-a_{0}}<\frac{K_{2}a_{2}}{m_{2}-a_{2}}\;\text{or}\; \end{array}$$

(52) $$\begin{array}{c} \frac{L_{0}a_{0}}{m_{0}-a_{0}}=\frac{K_{2}a_{2}}{m_{2}-a_{2}}\;\text{and}\;\frac{L_{0}m_{0}}{{\left(m_{0}-a_{0}\right)}^{2}}<\frac{K_{2}m_{2}}{{\left(m_{2}-a_{2}\right)}^{2}} \end{array}$$

The cases (c) or (d) occur if and only if $s_{2}^{0}\left(0\right)>M_{2}\left(0\right)$ holds or $s_{2}^{0}\left(0\right)=M_{2}\left(0\right)$ and $\frac{ds_{2}^{0}}{dD}\left(0\right)>\frac{dM_{2}}{dD}\left(0\right)$ hold, viz.

(53) $$\begin{array}{c} \frac{L_{0}a_{0}}{m_{0}-a_{0}}>\frac{K_{2}a_{2}}{m_{2}-a_{2}}\;\text{or}\; \end{array}$$

(54) $$\begin{array}{c} \frac{L_{0}a_{0}}{m_{0}-a_{0}}=\frac{K_{2}a_{2}}{m_{2}-a_{2}}\;\text{and}\;\frac{L_{0}m_{0}}{{\left(m_{0}-a_{0}\right)}^{2}}>\frac{K_{2}m_{2}}{{\left(m_{2}-a_{2}\right)}^{2}} \end{array}$$

Notice that it is easy to make the difference between case (c) and case (d): the first occurs when $M_{2}\left(D_{1}\right)<s_{2}^{0}\left(D_{1}\right)$ and the second when $M_{2}\left(D_{1}\right)>s_{2}^{0}\left(D_{1}\right)$. Since *D*_1_ is the positive solution of the algebraic quadratic equation $s_{2}^{0}\left(D\right)=s_{2}^{1}\left(D\right),$ it is possible to have an expression for *D*_1_ with respect to the biological parameters. However, this is a complicated expression involving many parameters and the preceding conditions $M_{2}\left(D_{1}\right)<s_{2}^{0}\left(D_{1}\right)$ or $M_{2}\left(D_{1}\right)>s_{2}^{0}\left(D_{1}\right)$ have no biological interpretation. We simply remark here that the function $s_{2}^{0}\left(D\right)$ has a vertical asymptote for $D=m_{0}-a_{0}$ and the function *M*_2_(*D*) has a vertical asymptote for $D=m_{2}-a_{2}$. Therefore, if $m_{0}-a_{0}<m_{2}-a_{2}$ then case (c) occurs, so that a necessary (but not sufficient) condition for case (d) to occur is $m_{0}-a_{0}>m_{2}-a_{2}$. If *m*_2_ is sufficiently small then case (d) can occur.

The observations from the numerical analysis suggest that the role of the chlorophenol degrader as a secondary hydrogen scavenger is critical in maintaining full chlorophenol mineralisation and system stability, particularly at higher dilution rates, as shown by comparing cases (c) and (d) . More significantly, the results coupled with the parameter relationships shown in ((51), (52), (53), (54)), highlight the necessary conditions under which the ideal case (SS3 stable) is achieved and, in general, this is a coupling of the two key parameters describing the half-saturation constant and maximum specific growth rates between the two hydrogen competitors.

## 10 Conclusions

In this work we have generalised a simplified mechanistic model describing the anaerobic mineralisation of chlorophenol in a two-step food-web. We give conditions for the existence and stability of the steady-states in the case that maintenance is excluded from the system. However, with a decay term present, purely analytical determination of stability was not achievable.

We confirm the findings of previous numerical analysis by [24] that with chlorophenol as the sole input substrate, three steady-states are possible. However, the analysis goes further and we determine that under certain operating conditions, two of these steady-states (SS2 and SS3) can become stable, whilst SS1 always exists and is always stable. Furthermore, without maintenance we can explicitly determine the stability of the system, and form analytical expressions of the boundaries between the different stability regions.

As the boundary of $J_{3}$ is not open to analytical determination in the case with maintenance, we determined numerically (substituting the general growth function with the classical Monod-type growth kinetics) the existence and stability of the system over a range of practical operating conditions (dilution rate and chlorophenol input). For comparison and confirmation, we also performed this for the case without maintenance and found the same regions in both cases, with variations only in their shape and extent. For example, whilst the boundary between $J_{1}$ and $J_{4}$ terminates at the origin without maintenance, with maintenance it is located at *F*_1_(0)/*Y*_3_*Y*_4_ ≈ 0.0195. More interestingly, the addition of a decay term results in an extension of the SS3 unstable steady-state, reducing the potential for successful chlorophenol demineralisation at relatively low dilution rates and substrate input concentrations. Additionally, we show that at the boundary between $J_{3}$ and $J_{5},$ there is numerical evidence of Hopf bifurcation occurring and that a limit cycle in SS3 emerges.

Finally, we gave an example of how the model could be used to probe the system to answer specific questions regarding model parameterisation. Here we have indicated that a switch in dominance between two organisms competing for hydrogen results in the system becoming unstable and a loss in viability. This is perhaps intuitive to microbiologists, but here it has been proven using mathematical analysis and could be used to determine critical limits of the theoretical parameter values in shifting between a stable and unstable system. Whilst parameters are not arbitrary in reality, the potential for microbial engineering or synthetic biology to manipulate the properties of organisms makes this observation all the more pertinent.

## Figures and Tables

**Fig. B.14:**
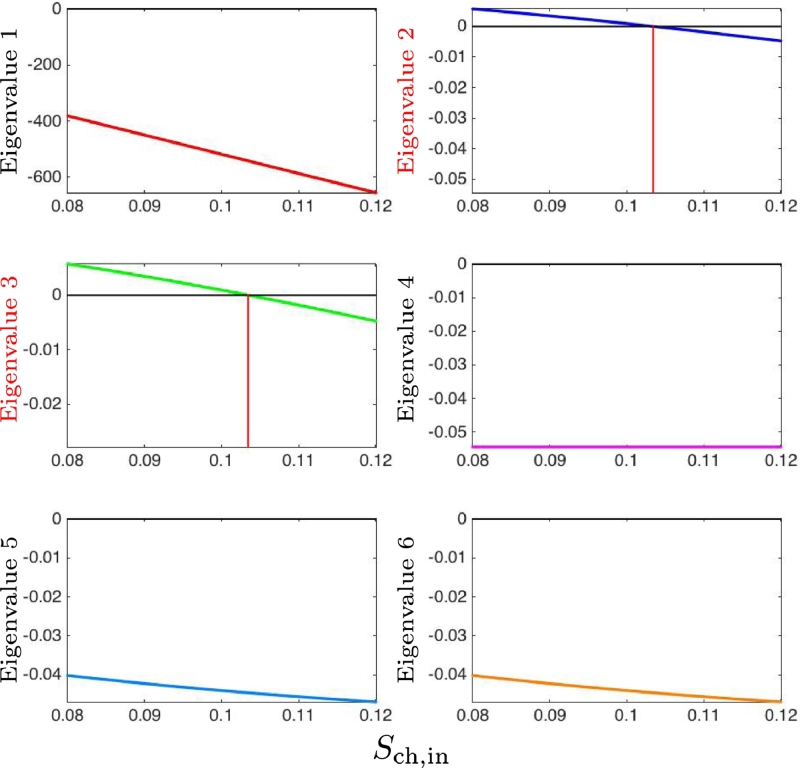
Real parts of the eigenvalues determined at D=0.01 and $S_{\text{ch},\text{in}}=\left[0.08,0.12\right],$ in the case with maintenance. The red vertical lines indicate the location where the eigenvalue crosses zero. (For interpretation of the references to colour in this figure legend, the reader is referred to the web version of this article.)

## Appendix A Numerical methods

We consider sets of operating parameters (*D* and *S*_ch, in_) for each of the three steady-states, and using Matlab, the complex polynomials for each steady-state can be solved by substitution of parameter values (see Table 1) into the explicit solution. By investigating the signs of the solutions and the eigenvalues, respectively we determine which steady-states are meaningful and stable. By exploring a localised region of suitable operating parameters, we then generate a phase plot showing where each steady-state is stable, bistable or unstable.

## Appendix B Numerical evidence for Hopf Bifurcation

In Section 8, we show the operating diagrams with the parameters given in Table 1, and determine numerically that as the parameter *S*_ch, in_ increases at a fixed dilution rate ($D=0.01\;d^{-1}$), the system bifurcates through several stability domains. We claim that as we cross the boundary between regions $J_{5}$ and $J_{3},$ we observe a Hopf bifurcation, and, in $J_{5},$ close to the boundary with $J_{3},$ a limit cycle appears. In order to test this numerically, we checked the real parts of the six eigenvalues at each point along the transect shown in Fig 9 (10000 points in total), and plotted their values. Fig B.14 indicates the conditions for a Hopf bifurcation are satisfied as eigenvalues 2 and 3 both change their sign when passing through the coordinate (0, 0.1034) and the real part of all eigenvalues 1, 4 and 6 remain negative.

## Appendix C Proofs

In this Section we give the proofs of the results.

### C1 General case

In these proofs, we do not assume that the growth function are of Monod type (Eq. 23). We only assume that the growth functions satisfy **H1**–**H8**.

#### Proof of Lemma 1

Let *s*_2_ ≥ 0 be fixed. By **H4**, the function

$$s_{0}\in \left[0,+\infty \right)\mapsto \mu_{0}\left(s_{0},s_{2}\right)\in \left[0,\mu_{0}\left(+\infty ,s_{2}\right)\right)$$

is monotonically increasing. Hence, it has an inverse function denoted by:

$$y\in \left[0,\mu_{0}\left(+\infty ,s_{2}\right)\right)\mapsto M_{0}\left(y,s_{2}\right)\in \left[0,+\infty \right),$$

such that for all *s*_0_ ≥ 0, *s*_2_ ≥ 0 and $y\in [0,\mu_{0}\left(+\infty ,s_{2}\right))$ (Eq. 25) holds.

The proofs for the existence of *M*_1_ and *M*_2_ are similar and are left to the reader.

### C2 Existence of steady-states

#### Proof of Lemma 2

Assume first that $x_{0}=0$. Then, as a consequence of (Eq. 38), $s_{0}=s_{0}^{\text{in}}$ and, as a consequence of (Eq. 39), we have:

$$Ds_{1}+\mu_{1}\left(s_{1},s_{2}\right)x_{1}=0$$

which implies $s_{1}=0$ and $\mu_{1}\left(s_{1},s_{2}\right)x_{1}=0$. Therefore, as a consequence of (Eq. 36) we have $x_{1}=0$. Replacing $x_{0}=0$ and $x_{1}=0$ in (Eq. 40), we have:

$$Ds_{2}+\mu_{2}\left(s_{2}\right)x_{2}=0$$

which implies $s_{2}=0$ and $\mu_{2}\left(s_{2}\right)x_{2}=0$. Therefore, as a consequence of (Eq. 37), $x_{2}=0$. Hence, the steady-state is SS1.

Assume now that $x_{1}=0$. Then, as a consequence of (Eq. 40), we have:

$$Ds_{2}+\omega \mu_{0}\left(s_{0},s_{2}\right)x_{0}+\mu_{2}\left(s_{2}\right)x_{2}=0$$

which implies $s_{2}=0,$$\mu_{0}\left(s_{0},s_{2}\right)x_{0}=0$ and $\mu_{2}\left(s_{2}\right)x_{2}=0$. Therefore, as a consequence of (Eq. 35), $x_{0}=0$. As shown previously this implies that the steady-state is SS1. Evaluated at SS1 the Jacobian matrix of ((14), (15), (16), (17), (18), (19)) is:

$$\left[\begin{array}{cccccc} -D-a_{0} & 0 & 0 & 0 & 0 & 0\\ 0 & -D-a_{1} & 0 & 0 & 0 & 0\\ 0 & 0 & -D-a_{2} & 0 & 0 & 0\\ 0 & 0 & 0 & -D & 0 & 0\\ 0 & 0 & 0 & 0 & -D & 0\\ 0 & 0 & 0 & 0 & 0 & -D \end{array}\right]$$

Thus, SS1 is stable.

#### Proof of Lemma 3

Since *x*_0_ > 0 and *x*_1_ > 0, then, as a consequence of (Eqs. 35, 36), we have:

$$\mu_{0}\left(s_{0},s_{2}\right)=D+a_{0},\;\mu_{1}\left(s_{1},s_{2}\right)=D+a_{1}$$

Hence, using (Eqs. 25, 26), we have:

(C.1) $$s_{0}=M_{0}\left(D+a_{0},s_{2}\right),\;s_{1}=M_{1}\left(D+a_{1},s_{2}\right)$$

Using (Eqs. 38, 39), we have (Eq. 42). Using (Eq. 40) we obtain:

(C.2) $$-s_{2}+\left(s_{0}^{\text{in}}-s_{0}-s_{1}\right)-\omega \left(s_{0}^{\text{in}}-s_{0}\right)=0$$

If *ω* ≥ 1 this equation has no solution. If *ω* < 1 this equation is equivalent to:

$$s_{0}^{\text{in}}=s_{0}+\frac{s_{1}+s_{2}}{1-\omega }.$$

Using (Eq. C.1) we see that *s*_2_ must be a solution of (Eq. 41). Since *s*_1_ > 0 and *s*_2_ > 0 then, from (Eq. C.2) we have necessarily:

$$s_{1}+s_{2}=\left(1-\omega \right)\left(s_{0}^{\text{in}}-s_{0}\right)>0$$

so that $s_{0}^{\text{in}}-s_{0}>0$. From (Eq. 42) we deduce that *x*_0_ > 0. Since $s_{0}^{\text{in}}-s_{0}>0$ and *s*_2_ > 0 then, from (Eq. C.2) we have necessarily:

$$\omega \left(s_{0}^{\text{in}}-s_{0}\right)+s_{2}=s_{0}^{\text{in}}-s_{0}-s_{1}>0$$

so that $s_{0}^{\text{in}}-s_{0}-s_{1}>0$ From (Eq. 42) we deduce that *x*_1_ > 0.

#### Proof of Lemma 4

Since *x*_0_ > 0, *x*_1_ > 0 and *x*_2_ > 0, then, as a consequence of ((35), (36), (37)), we obtain:

$$\mu_{0}\left(s_{0},s_{2}\right)=D+a_{0},\;\mu_{1}\left(s_{1},s_{2}\right)=D+a_{1},\;\mu_{2}\left(s_{2}\right)=D+a_{2}$$

Hence, using ((25), (26), (27)), *s*_0_, *s*_1_ and *s*_2_ are given by (Eq. 43). Using ((38), (39), (40)) we obtain (Eq. 44). For *x*_2_ to be positive it is necessary that *s*_0_, *s*_1_ and *s*_2_ satisfy the condition:

(C.3) $$\left(1-\omega \right)\left(s_{0}^{\text{in}}-s_{0}\right)>s_{1}+s_{2},$$

If *ω* ≥ 1 this equation has no solution. If *ω* < 1 this equation is equivalent to the condition:

$$s_{0}^{\text{in}}>s_{0}+\frac{s_{1}+s_{2}}{1-\omega }.$$

Using (Eq. 43), this condition is the same as

$$s_{0}^{\text{in}}>\psi \left(M_{2}\left(D+a_{2}\right)\right)=F_{2}\left(D\right)$$

Therefore, from (Eq. C.3) we have $s_{0}^{\text{in}}-s_{0}>0$ and $s_{0}^{\text{in}}-s_{0}-s_{1}>0,$ so that *x*_0_ > 0 and *x*_1_ > 0.

### C3 Stability of steady-states

We use the change of variables:

(C.4) $$z_{0}=s_{0}+x_{0},\;z_{1}=s_{1}+x_{1}-x_{0},\;z_{2}=s_{2}+x_{2}+\omega x_{0}-x_{1}$$

Therefore, (Eqs. 14–19), with $a_{0}=a_{1}=a_{2}=0,$ become:

(C.5) $$\begin{array}{cc} \frac{dx_{0}}{dt} & =-Dx_{0}+\mu_{0}\left(z_{0}-x_{0},z_{2}-\omega x_{0}+x_{1}-x_{2}\right)x_{0} \end{array}$$

(C.6) $$\begin{array}{cc} \frac{dx_{1}}{dt} & =-Dx_{1}+\mu_{1}\left(z_{1}+x_{0}-x_{1},z_{2}-\omega x_{0}+x_{1}-x_{2}\right)x_{1} \end{array}$$

(C.7) $$\begin{array}{cc} \frac{dx_{2}}{dt} & =-Dx_{2}+\mu_{2}\left(z_{2}-\omega x_{0}+x_{1}-x_{2}\right)x_{2} \end{array}$$

(C.8) $$\begin{array}{cc} \frac{dz_{0}}{dt} & =D\left(s_{0}^{\text{in}}-z_{0}\right) \end{array}$$

(C.9) $$\begin{array}{cc} \frac{dz_{1}}{dt} & =-Dz_{1} \end{array}$$

(C.10) $$\begin{array}{cc} \frac{dz_{2}}{dt} & =-Dz_{2} \end{array}$$

In the variables (*x*_0_, *x*_1_, *x*_2_, *z*_0_, *z*_1_, *z*_2_) where *z*_0_, *z*_1_ and *z*_2_ are defined by (Eq. C.4), the steady-states SS1, SS2 and SS3 are given by:

1. $\text{SS}1=(0,0,0,s_{0}^{\text{in}},0,0)$
2. $\text{SS}2=(x_{0},x_{1},0,s_{0}^{\text{in}},0,0),$ where *x*_0_ and *x*_1_ are defined by (Eq. 47).
3. $\text{SS}3=(x_{0},x_{1},x_{2},s_{0}^{\text{in}},0,0),$ where *x*_0_, *x*_1_ and *x*_2_ are defined by (Eq. 48).

Let $\left(x_{0},x_{1},x_{2},s_{0}^{\text{in}},0,0\right)$ be a steady-state. The Jacobian matrix of (Eqs. C.5–C.10) has the block triangular form:

$$J=\left[\begin{array}{cc} J_{1} & J_{2}\\ 0 & J_{3} \end{array}\right]$$

where

$$J_{1}=\left[\begin{array}{ccc} \mu_{0}-D-\left(E+\omega F\right)x_{0} & Fx_{0} & -Fx_{0}\\ \left(G+\omega H\right)x_{1} & \mu_{1}-D-\left(G+H\right)x_{1} & Hx_{1}\\ -\omega Ix_{2} & Ix_{2} & \mu_{2}-D-Ix_{2} \end{array}\right]$$

$$J_{2}=\left[\begin{array}{ccc} Ex_{0} & 0 & Fx_{0}\\ 0 & Gx_{1} & -Hx_{1}\\ 0 & 0 & Ix_{2} \end{array}\right],\;J_{3}=\left[\begin{array}{ccc} -D & 0 & 0\\ 0 & -D & 0\\ 0 & 0 & -D \end{array}\right]$$

and

$$E=\frac{\partial \mu_{0}}{\partial s_{0}},\;F=\frac{\partial \mu_{0}}{\partial s_{2}},\;G=\frac{\partial \mu_{1}}{\partial s_{1}},\;H=-\frac{\partial \mu_{1}}{\partial s_{2}},\;I=\frac{d\mu_{2}}{ds_{2}}$$

are evaluated at the steady-state.

Since **J** is a block triangular matrix, its eigenvalues are −D (with multiplicity 3) together with the eigenvalues of the 3 × 3 upper-left matrix **J_1_**. Note that we have used the opposite sign for the partial derivative $H=-\partial \mu_{1}/\partial s_{2},$ so that all constants involved in the computation become positive, which will simplify the analysis of the characteristic polynomial of **J_1_**.

#### Proof of Proposition 2

Evaluated at SS2, the matrix **J_1_** is:

$$J_{1}=\left[\begin{array}{ccc} -\left(E+\omega F\right)x_{0} & Fx_{0} & -Fx_{0}\\ \left(G+\omega H\right)x_{1} & -\left(G+H\right)x_{1} & Hx_{1}\\ 0 & 0 & \mu_{2}-D \end{array}\right]$$

Since **J_1_** is a block triangular matrix, its eigenvalues are simply $\mu_{2}-D,$ together with the eigenvalues of the 2 × 2 upper-left matrix. Note that the trace of this 2 × 2 matrix is negative. Hence, its eigenvalues are of negative real part if, and only if, its determinant is positive, that is if, and only if,

(C.11) $$\begin{array}{c} E(G+H)-(1-\omega )FG>0 \end{array}$$

Using

$$\frac{\partial M_{0}}{\partial s_{2}}=-\frac{\partial \mu_{0}}{\partial s_{2}}{\left[\frac{\partial \mu_{0}}{\partial s_{0}}\right]}^{-1}=-F/E$$

$$\frac{\partial M_{1}}{\partial s_{2}}=-\frac{\partial \mu_{1}}{\partial s_{2}}{\left[\frac{\partial \mu_{1}}{\partial s_{1}}\right]}^{-1}=H/G$$

we deduce from

$$\psi \left(s_{2}\right)=M_{0}\left(D,s_{2}\right)+\frac{M_{1}\left(D,s_{2}\right)+s_{2}}{1-\omega }$$

that

$$\frac{\partial \psi }{\partial s_{2}}=\frac{\partial M_{0}}{\partial s_{2}}+\frac{\frac{\partial M_{1}}{\partial s_{2}}+1}{1-\omega }=-\frac{F}{E}+\frac{\frac{H}{G}+1}{1-\omega }$$

Hence,

(C.12) $$\frac{\partial \psi }{\partial s_{2}}=\frac{E(G+H)-(1-\omega )FG}{(1-\omega )EG}$$

Therefore, the condition of stability, (Eq. C.11), is equivalent to $\frac{\partial \psi }{\partial s_{2}}>0$. Hence, we have proven that SS2 is stable if, and only if, *μ*_2_(*s*_2_) < *D* and $\frac{\partial \psi }{\partial s_{2}}>0$.

#### Proof of Proposition 3

Evaluated at SS3, the matrix **J_1_** is:

$$J_{1}=\left[\begin{array}{ccc} -\left(E+\omega F\right)x_{0} & Fx_{0} & -Fx_{0}\\ \left(G+\omega H\right)x_{1} & -\left(G+H\right)x_{1} & Hx_{1}\\ -\omega Ix_{2} & Ix_{2} & -Ix_{2} \end{array}\right]$$

The characteristic polynomial is given by:

(C.13) $$\begin{array}{c} \lambda^{3}+f_{2}\lambda^{2}+f_{1}\lambda +f_{0}=0 \end{array}$$

where

(C.14) $$\begin{array}{cc} f_{2} & =Ix_{2}+\left(G+H\right)x_{1}+\left(E+\omega F\right)x_{0} \end{array}$$

(C.15) $$\begin{array}{cc} f_{1} & =\Delta x_{0}x_{1}+EIx_{0}x_{2}+GIx_{1}x_{2} \end{array}$$

(C.16) $$\begin{array}{cc} f_{0} & =EGIx_{0}x_{1}x_{2} \end{array}$$

and Δ=E(G+H)−(1−ω)FG.

To satisfy the Routh-Hurwitz criteria, we require *f_i_* > 0, for i=0,1,2 and $f_{1}f_{2}-f_{0}>0$. Notice that:

(C.17) $$f_{1}f_{2}-f_{0}=\left(EIx_{0}x_{2}+\Delta x_{0}x_{1}\right)f_{2}+\left(Ix_{2}+\left(G+H\right)x_{1}+\omega Fx_{0}\right)GIx_{1}x_{2}$$

We always have *f*_0_ > 0 and *f*_2_ > 0.

From (Eq. C.12) we deduce that $\Delta =\left(1-\omega \right)EG\frac{\partial \psi }{\partial s_{2}}$ . Therefore, if *F*_3_(*D*) ≥ 0, viz. $\frac{\partial \psi }{\partial s_{2}}\geq 0,$ then *Δ* ≥ 0. Hence, *f*_1_ > 0 and $f_{1}f_{2}-f_{0}>0,$ so that SS3 is stable.

On the other hand, if $\frac{\partial \psi }{\partial s_{2}}<0$ and *x*_2_ is very small, which occurs when SS3 is very close to SS2^♭^, then *f*_2_ has the sign of *Δ* since the term with *x*_2_ is negligible compared to the term *Δx*_0_*x*_1_:

$$f_{1}=\Delta x_{0}x_{1}+\left(EIx_{0}+GIx_{1}\right)x_{2}<0$$

Thus, SS3 is unstable.

#### Proof of Proposition 4

Since we always have *f*_0_ > 0 and *f*_2_ > 0, from the previous proof it follows that SS3 is stable if, and only if, $f_{1}f_{2}-f_{0}>0$. Indeed, this condition implies that we have also *f*_1_ > *f*_0_/*f*_2_ > 0. Using (Eq. 49) and (Eq. C.17), we see that:

$$f_{1}f_{2}-f_{0}=F_{4}\left(D,s_{0}^{\text{in}}\right)$$

Therefore, the condition $f_{1}f_{2}-f_{0}>0$ is equivalent to $F_{4}\left(D,s_{0}^{\text{in}}\right)>0$.

### C4 Operating diagrams

#### Proof of Proposition 5

We know that SS1 always exists and is stable. We know that SS2^♭^ is unstable if it exists. The curves *Γ*_1_ and *Γ*_2_ of the operating diagram, given by (Eq. 45) and (Eq. 46), respectively are defined for *D* < *D*_1_ and *D* < *D*_2_, respectively and they are tangent for $D=D_{3},$ where $D_{1}=0.452,$$D_{2}=0.393$ and $D_{3}=0.078$. Therefore *F*_3_(*D*) < 0 for *D* < *D*_3_ and *F*_3_(*D*) > 0 for *D* > *D*_3_. Using Table 7 and Remark 2, we obtain the following results:

• $J_{1}$ is defined by *D* ≥ *D*_1_ or 0 < *D* < *D*_1_ and *S*_ch, in_ < *F*_1_(*D*)/*Y*_3_*Y*_4_. Therefore, SS1 is the only existing steady-state in this region.
• $J_{2}$ is defined by *D*_3_ < *D* < *D*_1_ and *F*_1_(*D*)/*Y*_3_*Y*_4_ < *S*_ch, in_ < *F*_2_(*D*)/*Y*_3_*Y*_4_. Therefore, both steady-state SS2 exist and SS2^♯^ is stable since *F*_3_(*D*) > 0.
• $J_{3}$ is defined by 0 < *D* < *D*_2_ and *F*_2_(*D*)/*Y*_3_*Y*_4_ < *S*_ch, in_, and *F*_4_(*D, Y*_3_*Y*_4_*S*_ch, in_) > 0 when 0 < *D* < *D*_3_. Therefore, SS3 exists and is stable, both steady-state SS2 exist and SS2^♯^ is unstable since *F*_3_(*D*) < 0.
• $J_{4}$ is defined by 0 < *D* < *D*_3_ and *F*_1_(*D*)/*Y*_3_*Y*_4_ < *S*_ch, in_ < *F*_2_(*D*)/*Y*_3_*Y*_4_. Therefore, both steady-state SS2 exist and SS2^♯^ is unstable since *F*_3_(*D*) < 0.
• $J_{5}$ is defined by 0 < *D* < *D*_3_, *F*_2_(*D*)/*Y*_3_*Y*_4_ < *S*_ch, in_, and *F*_4_(*D, Y*_3_*Y*_4_*S*_ch, in_) < 0. Therefore, SS3 exists and is unstable and both steady-state SS2 exist and SS2^♯^ is unstable since *F*_3_(*D*) < 0.

#### Proof of Propositions 6–8

The result follows from Table 7 and Remark 2. The details are as in the proof of Proposition 5.
